# A treatment‐specific marginal structural Cox model for the effect of treatment discontinuation

**DOI:** 10.1002/pst.2211

**Published:** 2022-03-31

**Authors:** Dana Johnson, Karen Pieper, Shu Yang

**Affiliations:** ^1^ Biostatistics United Therapeutics Corp. Silver Spring North Carolina USA; ^2^ Statistics Thrombosis Research Institute London UK; ^3^ Department of Statistics North Carolina State University Raleigh North Carolina USA

**Keywords:** articifial censoring, causal inference, discontinuation, inverse probability weighting, time‐dependent confounding

## Abstract

Patients taking a prescribed medication often discontinue their treatment; however, this may negatively impact their health outcomes. If doctors had statistical evidence that discontinuing some prescribed medication shortened, on average, the time to a clinical event (e.g., death), they could use that knowledge to encourage their patients to stay on the prescribed treatment. We describe a treatment‐specific marginal structural Cox model for estimation of the causal effect of treatment discontinuation on a survival endpoint. The effect of treatment discontinuation is quantified by the hazard ratio of the event hazard rate had the population followed the regime “take treatment a until it is discontinued at some time ν,” versus the event hazard rate had the population never discontinued treatment a. Valid causal analysis requires control for treatment confounding, regime confounding, and censoring due to regime violation. We propose new inverse probability of regime compliance weights to address the three issues simultaneously. We apply the framework to data from the Global Anticoagulant Registry in the FIELD–Atrial Fibrillation (GARFIELD‐AF) study. Patients from this study are treated with one of two types of oral anticoagulants (OACs). We test whether the causal effect of treatment discontinuation differs by type of OAC, and we also estimate the size and direction of the effect. We find evidence that OAC discontinuation increases the hazard for certain events, but we do not find evidence that this effect differs by treatment.

## 1 INTRODUCTION

Atrial fibrillation (AF) is a the most common type of cardiac arrhythmia. Having this condition increases, by more than fivefold, the risk for clinical events such as ischemic stroke and blood clots.^1^, ^2^ In order to prevent these events from happening to individuals with AF, clinicians may prescribe these patients a blood thinner, also known as an oral anticoagulant (OAC). There are two main classes of OAC—vitamin K antagonists (VKA) and non‐vitamin K oral anticoagulants (NOACs), with NOACs being the newer of these two classes.^3^, ^4^ The VKAs and the NOACs act differently within the body. Specifically, VKAs such as Warfarin inhibit the synthesis of various clotting factors, whereas NOACs such as dabigatran and rivaroxaban directly target a particular clotting factor.^4^ Despite their potential to prevent stroke and clotting,^4^ high levels of both VKA and NOAC treatment discontinuation (some has high as 53%) have been reported in the AF population.^5^, ^6^, ^7^ This has sparked an interest in studying the causal effect of OAC discontinuation on endpoints such as stroke and death. Furthermore, because VKAs and NOACs work differently to reduce the risk of stroke, it is important to examine whether or not the effect of OAC discontinuation differs by class of OAC.

The Global Anticoagulant Registry in the FIELD–Atrial Fibrillation (GARFIELD‐AF) study contains data on patients recently diagnosed with nonvalvular atrial fibrillation. Recruitment for the prospective study began in 2009 and was completed in 2016. Each patient in the study had at least one risk factor for stroke and agreed to 2 years of follow‐up.^8^, ^9^ A rich set of baseline information was collected on each patient, including: age, gender, race, and medical history (e.g., hypertension, diabetes, heart failure, bleeding history). Time‐varying information was also collected on each patient. This included if and when a patient had a stroke, myocardial infarction (MI), left atrial appendage procedure (LAAP),^10^ or various bleeding events (e.g., minor bleed; major bleed; nonmajor, clinically relevant bleed) as well as if and when a patient discontinued treatment. Refer to Kakkar et al.^8^ for additional details on the information obtained throughout the study. We focus on the 23,882 patients from cohorts 3–5, whose initial treatment was either VKA or NOAC. Interest lies in estimating the causal effect of OAC discontinuation on the following endpoints: death, cardiovascular death, stroke/systemic embolism (SE), acute MI, as well as combinations of these endpoints.

**FIGURE 1:**
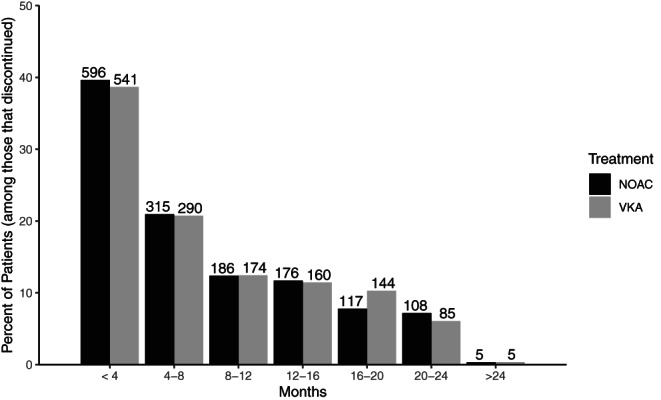
Months from start of treatment to discontinuation

According to the GARFIELD‐AF Steering Committee, most patients that do not permanently discontinue treatment, but instead temporarily get off treatment, are off treatment for just one to 7 days before getting back on. For this reason, we formally define treatment discontinuation as being off treatment for at least seven consecutive days. Under this definition, 2902 (1399 from VKA group; 1503 from NOAC group) of the 23,882 AF patients discontinued treatment during follow‐up, and prior to regime violation, which is discussed later in this section. In both the group of patients that started on VKA and the group of patients that started on NOAC, most patients that discontinued treatment did so early into follow‐up. Refer to Figure 1 for a visual representation of when (how long into follow‐up) the GARFIELD‐AF patients discontinued treatment.

While the time‐dependent (TD) Cox proportional hazards (PH) model is often used to analyze survival data with time‐varying covariates, it has been shown than in certain cases, using a TD Cox PH model to estimate the causal effect of treatment (or in this case, treatment discontinuation) on a given survival endpoint can yield biased estimates of the causal effect.^11^, ^12^ In particular, these issues arise when there exists a TD confounding variable such that past exposure predicts the TD confounder. We call this “regime confounding.” This is the case for the treatment discontinuation problem we consider. For example, consider the time‐varying information on major bleeding recorded in the GARFIELD‐AF study. The occurrence (or absence) of a major bleed is predictive of treatment discontinuation (or persistence), and it is prognostic for clinical endpoints such as death. Furthermore, whether or not an individual experiences a major bleed is influenced by the individual's previous choice to either stay on the drug or to discontinue it. Thus, analyzing the causal effect of OAC discontinuation in the GARFIELD‐AF patients warrants an advanced approach.

Structural failure time models^13^, ^14^ and marginal structural models (MSMs)^12^, ^15^ are two common approaches for estimating causal effects in the presence of TD confounders that are themselves affected by past exposure. MSMs are an appealing choice because they resemble standard models, unlike structural failure time models.^15^ This makes them easy to interpret. Yang et al.^16^ used MSMs to estimate the causal effect of treatment discontinuation on a survival endpoint. Specifically, they accomplished this by first casting the intervention, in this case treatment discontinuation, as a treatment policy of the form “take treatment until you discontinue treatment at time ν,” where ν is any positive real number. The authors then proposed using a dynamic regime MSM to estimate the population hazard rate of having a clinical event had all patients in the population followed the treatment policy dictated by “ν.” By computing the hazard ratio of the event hazard had all patients followed the time‐to‐discontinuation regime “ν,” relative to the event hazard had all patients remained on treatment (never discontinued), they were able to quantify the causal effect of treatment discontinuation on survival. While this approach allows intervention to be a function of the time‐to‐discontinuation time “ν,” it does not allow it to be a function of more than one treatment. In the context of fixed treatment regimes (as opposed to dynamic regimes, where the treatment policy takes into account each patient's evolving outcomes), we extend the framework in Yang et al.^16^ to the case where each patient's initial treatment is not necessarily the same. We do this by considering the following treatment‐specific time‐to‐discontinuation policy: “take treatment a (VKA or NOAC) until you discontinue treatment a at time *ν*”; however, the extension is not straightforward. Yang et al.^16^ took for granted that a patient will neither switch treatments throughout follow up nor get back on a treatment once he/she has discontinued treatment. If either of these two events *do* occur, the treatment‐specific time‐to‐discontinuation regime that we are interested in is violated. 3100 (1738 from VKA group; 1362 from NOAC group) of the patients in the GARFIELD‐AF data have violated the regime in one of these two ways. Two naive approaches to account for the issue of regime violation are to (1) ignore when a patient has violated the regime and use the initial treatment (VKA/NOAC) to determine which treatment group the patient belongs to or (2) completely remove patients that violate the regime from the analysis. Unfortunately, both of these solutions potentially bias the analysis. If the first approach were taken, patients from both OAC groups would be used to represent a single OAC group–contaminating the desired treatment‐specific analysis. Additionally, any potential effect of discontinuation on survival may be weakened because patients that discontinued treatment, but then later got back on treatment, would still be included in the group of patients that discontinued. If the second approach is taken, the resulting sample may not be representative of the population–biasing results. This would be the case if patients that violate the regime are systematically different than patients who do not. In this paper, we address regime violation by artificially censoring patients when they violate the treatment‐specific time‐to‐discontinuation regime. Artificially censoring patients in this way may induce informative censoring, so it must be appropriately accounted for in the analysis. We propose inverse probability of regime compliance (IPRC) weights to appropriately adjust for artificially censoring these patients, as well as to adjust for any treatment confounding and regime confounding that may exist. We apply this method to the GARFIELD‐AF data in order to test whether the effect of OAC discontinuation differs by treatment and to estimate the (potentially treatment‐specific) effect of OAC discontinuation.

The remainder of this paper is organized as follows. Section 2 introduces the statistical framework used throughout the paper. This includes the notation, the formal specification of the causal parameter of interest, assumptions, and the treatment regime MSM for the hazard of treatment discontinuation. In Section 3, unbiased estimating equations for estimation of the treatment regime MSM and the asymptotic behavior of these estimators is discussed. The performance of the proposed IPRC weighted estimator is evaluated and compared against two naive estimation methods via simulation studies, and then in Section 4 the IPRC weighted estimator for $\beta_{a}^{*}$ is applied to the GARFIELD‐AF data. The results of this analysis are discussed in Sections 4.2 and 4.3. We end with a brief conclusion.

## 2 STATISTICAL FRAMEWORK

### 2.1 Notation

Suppose a sample of patients initiated on one of two treatment options are followed over time, where it is possible that some patients discontinue their treatment during follow up. Suppose further that the endpoint of interest is a clinically relevant failure time (e.g., time‐to‐death), which may be censored for certain individuals. Let $X_{i}$ denote the p×1 vector of observed baseline covariates for patient i, and let $A_{i}$ be his/her initial treatment assignment, where $A_{i}\in \left\{0,1\right\}$. For the GARFIELD‐AF analysis, we set $A_{i}=0$ if VKA is the initial treatment and $A_{i}=1$ if NOAC is the initial treatment. Let $T_{i}$ and $C_{i}$ be patient *i*'s possibly unobserved failure time and time‐to‐censoring, respectively, and let $U_{i}=\min\;\left(T_{i},C_{i}\right)$. Define the indicator function $1\left(\cdot \right)$ such that $1\left(B\right)=1$ if B is true and $1\left(B\right)=0$ otherwise, so that $\Delta_{i}=1\left(T_{i}\leq C_{i}\right)$ is the indicator that the clinical outcome was observed for patient i. Let $D_{i}$ be patient i's potentially unobserved time‐to‐treatment‐discontinuation, and let $V_{i}=\min\;\left(D_{i},U_{i}\right)$ so that $\Gamma_{\mathrm{Vi}}=1\left(D_{i}\leq U_{i}\right)$ is the indicator that patient i discontinued treatment prior to the clinical event and censoring. Define the time‐dependent discontinuation indicator at time t to be $Z_{V_{i}}\left(t\right)=1\left(V_{i}\leq t\right)\Gamma_{\mathrm{Vi}}$.

Additionally, let $Q_{i}\left(l\right)$ be a q×1 vector of all time‐dependent covariates, excluding $Z_{V_{i}}\left(l\right)$, that are observed for patient i at time l as long as he/she is still at risk at time l. The collection of these time‐dependent covariate vectors available on patient i at time t is then denoted ${\bar{Q}}_{i}\left(t\right)=\left\{Q_{i}\left(l\right):l\leq t\right\}$, for $t\leq U_{i}$. Similarly, we let ${\bar{Z}}_{V_{i}}\left(t\right)=\left\{Z_{V_{i}}\left(t\right):l<t\right\}$ for $t\leq U_{i}$. Using the potential outcomes framework,^17^, ^18^ let $D_{i}^{\left(a\right)}$ be patient *i*'s time‐to‐discontinuation had he/she taken treatment a. Let $T_{i}^{\left(a,v\right)}$ be the failure time that would be observed had patient i taken treatment a and discontinued that treatment at time ν, where $\nu \in I\;R^{+}$, and let $T_{i}^{\left(a,\infty \right)}$ be the potential failure time had patient i never discontinued treatment a. Furthermore, define $Q_{i}^{\left(a,\nu \right)}\left(l\right)$ to be the q×1 vector of time‐dependent covariates, excluding the time‐dependent discontinuation indicator, that would be observed at time l had patient i been on treatment a until discontinuing treatment at time v. Similarly, the collection of time‐dependent covariate vectors that would be available on patient i at time t, under the treatment regime dictated by $\left(a,\nu \right)$, is denoted by ${\bar{Q}}_{i}^{\left(a,\nu \right)}\left(t\right)=\left\{Q_{i}^{\left(a,\nu \right)}:l\leq t\right\}$ for $t\leq T_{i}^{\left(a,\nu \right)}$.

Recall that there are two ways that a patient can become inconsistent with the regime “take treatment a until discontinuing a at time ν,” which we call “regime violation.” If a patient switches treatment, for example, goes from VKA to NOAC or vice versa, then he/she becomes inconsistent with the regime of interest at the time treatment is switched. Additionally, a patient becomes inconsistent with the regime of interest the moment he/she gets back on any drug (VKA or NOAC) after already having discontinued initial treatment. Let $R_{i}$ denote patient *i*'s potentially unobserved time‐to‐regime‐violation, and let $S_{i}=\min\;\left(R_{i},U_{i}\right)$. Then $\Gamma_{\mathrm{Si}}=1\left(R_{i}\leq U_{i}\right)$ is the indicator that patient i violated the regime of interest during follow up. When $\Gamma_{\mathrm{Si}}=1$, we artificially censor patient i at time $S_{i}$ due to regime violation. Let $R_{i}^{\left(a,\nu \right)}$ be the potential outcomes version of $R_{i}$, defined in the same manner as $T_{i}^{\left(a,\nu \right)}$ and $Q_{i}^{\left(a,\nu \right)}$.

We assume i=1,…,n independent and identically distributed copies of $O_{i}=\left\{X_{i},A_{i},U_{i},\Delta_{i},V_{i},\Gamma_{\mathrm{Vi}},S_{i},\Gamma_{\mathrm{Si}},{\bar{Q}}_{i}\left(U_{i}\right)\right\}$ are observed. We define the observed event counting process as $N_{i}\left(t\right)=1\left(U_{i}\leq t,\Delta_{i}=1\right)$ and the observed at‐risk process as $Y_{i}\left(t\right)=1\left(U_{i}\geq t\right)$. The event and at‐risk process under the policy dictated by $\left(a,\nu \right)$ are written as $N_{i}^{\left(a,\nu \right)}\left(t\right)$ and $Y_{i}^{\left(a,\nu \right)}\left(t\right)$, respectively.

### 2.2 Model and assumptions

Define the treatment‐regime‐specific hazard of failure

$$\lambda_{\mathrm{a\nu}}\left(t\right)=\lim_{h\to 0}h^{-1}\mathrm{pr}\left(t\leq T^{\left(a,\nu \right)}<t+h|T^{\left(a,\nu \right)}\geq t\right),$$

which is the hazard of failing had all patients followed the regime “take treatment a until discontinuing at time ν.” This is the causal parameter of interest. We assume censoring is non‐informative, which means that the censoring time is independent of the full set of potential variables. Under this assumption, we have

$$\lambda_{\mathrm{a\nu}}\left(t\right)=\lim_{h\to 0}h^{-1}\mathrm{pr}\left(t\leq U^{\left(a,\nu \right)}<t+h,\Delta^{\left(a,\nu \right)}=1|U^{\left(a,\nu \right)}\geq t\right),$$

where $\Delta^{\left(a,\nu \right)}=1\left(T^{\left(a,\nu \right)}\leq C\right)$. Section 5 provides a brief explanation on how to extend the proposed method to the case when censoring depends on the observed data. We consider the following treatment‐regime‐specific marginal structural Cox model for the causal parameter:

(1) $$\lambda_{\mathrm{a\nu}}\left(t\right)=\lambda_{00}\left(t\right)\;\exp\;\left\{\beta^{1*}a+\beta^{2*}z_{\nu }\left(t\right)+\beta^{3*}{\mathrm{az}}_{\nu }\left(t\right)\right\},$$

$$\equiv \lambda_{a0}\left(t\right)\;\exp\;\left\{\beta_{a}z_{v}\left(t\right)\right\},$$

where $\lambda_{a0}\left(t\right)=\lambda_{00}\left(t\right)\exp\left(\beta^{1*}a\right)$, $z_{\nu }\left(t\right)=1\left(\nu \leq t\right)$, and $\beta_{a}=\beta^{2*}+\beta^{3*}a$. Under the model given in (1), $\beta_{a}$ is the log of the relative hazard of failing had the population taken treatment A=a and discontinued treatment, compared to if the population had taken treatment A=a and stayed on treatment a (never discontinued). If the effect of discontinuation is modified by treatment, this difference in the effect of discontinuation on the log of the relative hazard of failure had the population taken NOAC, versus had the population taken VKA, is quantified by the parameter $\beta^{3*}$. Interest lies in testing whether the effect of treatment discontinuation on failure time differs by treatment (test $\beta^{3*}=0$) and in making inference on $\beta_{a}$.

Due to the fundamental problem of causal inference that at most only one potential outcome can be observed for a particular subject, the parameters in the MSM are not identifiable with observed data in general. In order to estimate $\beta ={\left(\beta^{1*},\beta^{2*},\beta^{3*}\right)}^{T}$ in (1), we make three additional assumptions. First, we make the consistency assumption,^18^, ^19^ which states that the observed data are equal to the corresponding potential outcomes under the treatment regime that was actually followed. Specifically, if patient i followed the regime “take treatment a until discontinuing ν days after starting treatment”, we assume that ${\bar{Q}}_{i}\left(U_{i}\right),T_{i}$, and $R_{i}$ are equal to ${\bar{Q}}^{\left(a,v\right)}\left(U^{\left(a,v\right)}\right),T_{i}^{\left(a,\nu \right)}$, and $R_{i}^{\left(a,\nu \right)}$, respectively. For the counting process and at‐risk process, the consistency assumption implies that $N_{i}\left(t\right)=N_{i}^{\left(A_{i},V_{i}\right)}\left(t\right)$ and $Y_{i}\left(t\right)=Y_{i}^{\left(A_{i},V_{i}\right)}\left(t\right)$ for all t when $\Gamma_{\mathrm{Vi}}=1$. Note also that we apply the constraint that $N_{i}^{\left(a,\nu \right)}\left(t\right)=N_{i}^{\left(a,t\right)}\left(t\right)$ and $Y_{i}^{\left(a,\nu \right)}\left(t\right)=Y_{i}^{\left(a,t\right)}\left(t\right)$, for ν≥t. The full set of potential variables is denoted by

$$\begin{array}{l} F_{i}=\{X_{i},A_{i},C_{i},T_{i},D_{i},R_{i},{\bar{Q}}_{i}\left(T_{i}\right),{\bar{Z}}_{i}\left(T_{i}\right),(D_{i}^{\left(a\right)},T_{i}^{\left(a,\nu \right)},R_{i}^{\left(a,\nu \right)},{\bar{Q}}_{i}^{\left(a,\nu \right)}\left(T_{i}^{\left(a,\nu \right)}\right)\\ :\nu <T_{i}^{\left(a,\infty \right)},a\in \left\{0,1\right\})\}. \end{array}$$

We define $F_{\mathrm{Ai}}$ to be equal to $F_{i}$, excluding $A_{i}$; we define $F_{\mathrm{Di}}$ to be equal to $F_{i}$, excluding $D_{i}$ and $D_{i}^{\left(a\right)}$; and we define $F_{\mathrm{Ri}}$ to be equal to $F_{i}$, excluding $R_{i}$ and $\left\{R^{(a,\nu }:\nu <T^{\left(a,\infty \right)},a\in \left\{0,1\right\}\right\}$. We make the no unmeasured confounders assumption,^19^ under which the propensity score, the hazard of discontinuation, and the hazard of regime violation are independent of $F_{A},F_{D}$, and $F_{R}$, respectively, given the observed data available at time t. Thus, these three quantities are defined as follows. The propensity of receiving treatment a as the initial treatment is

$$\mathrm{pr}\left(A=a|F_{A}\right)=\mathrm{pr}\left(A=a|X\right)\equiv \pi \left(X\right),$$

the hazard of treatment discontinuation is
(2)

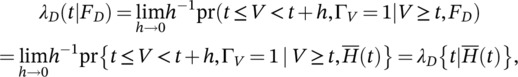

and the hazard of regime violation is
(3)

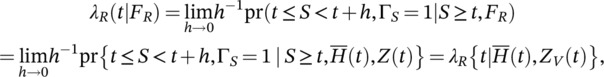

where $\bar{H}\left(t\right)=\left\{X,A,\bar{Q}\left(t\right)\right\}$. Using the definitions of the treatment discontinuation hazard and the regime violation hazard given in (2) and (3), respectively, we now define

$$K_{D}\left\{t|\bar{H}\left(t\right)\right\}=\exp\left[-\int_{0}^{t}\lambda_{D}\left\{l|\bar{H}\left(l\right)\right\}\mathrm{dl}\right],$$

$$f_{D}\left\{t|\bar{H}\left(t\right)\right\}=\lambda_{D}\left\{t|\bar{H}\left(t\right)\right\}K_{D}\left\{t|\bar{H}\left(t\right)\right\},$$

and

$$K_{R}\left\{t|\bar{H}\left(t\right),Z_{V}\left(t\right)\right\}=\exp\left[-\int_{0}^{t}\lambda_{R}\left\{l|\bar{H}\left(l\right),Z_{V}\left(l\right)\right\}\mathrm{dl}\right].$$

For an individual i, $K_{D}\left\{l|{\bar{H}}_{i}\left(l\right)\right\}$ and $K_{R}\left\{l|{\bar{H}}_{i}\left(l\right)\right\}$ can be viewed as the probability that individual i did not discontinue treatment before time l and the probability that individual i did not violate the posited treatment regime before time l, respectively. The quantity $f_{D}\left\{l|{\bar{H}}_{i}\left(l\right)\right\}$ can be interpreted as the probability that individual i discontinues treatment at some time within $\left[l,l+\mathrm{dl}\right]$. Finally, the positivity assumption^20^ we require is threefold. We require $\mathrm{pr}\left(A=a|X\right)>0$ for all x such that $\mathrm{pr}\left(X=x\right)>0$; for all $t,l,\bar{h}\left(l\right)$ such that $\mathrm{pr}\left\{\bar{H}\left(l\right)=\bar{h}\left(l\right),T=t,l\leq t\right\}>0,$ we require $f_{D}\left\{l|\bar{h}\left(l\right)\right\}$ > 0; and for all $t,l,\bar{h}\left(l\right),z\left(l\right)$ such that $\mathrm{pr}\left\{\bar{H}\left(l\right)=\bar{h}\left(l\right),Z_{V}\left(l\right)=z_{v}\left(l\right),T=t,l\leq t\right\}>0$, we require $\mathrm{pr}\left\{R\geq l|\bar{h}\left(l\right),z\left(l\right)\right\}>0$. The positivity assumption ensures that for each time point l for which a patient is still at risk for discontinuing an OAC, it is possible for that patient to follow any of the treatment‐discontinuation regimes still available at time l. It also ensures that the estimating equations given in Section 3 are well defined.

## 3 IDENTIFICATION AND PARAMETER ESTIMATION

### 3.1 Theory

Similar to Yang et al.,^16^ we define the following mean zero martingale process under the fixed treatment policy dictated by $\left(a,\nu \right)$: $M^{\left(a,\nu \right)}\left(t\right)=N^{\left(a,\nu \right)}\left(t\right)-\int_{0}^{t}\;\exp\;\left\{\beta_{a}z_{\nu }\left(l\right)\right\}Y^{\left(a,\nu \right)}\left(l\right)d\Lambda_{a0}\left(l\right)$, where $\Lambda_{a0}\left(l\right)$ is the cumulative baseline hazard at time l had the population taken treatment A=a. If the full set of potential variables were observed on each individual in the observed data, estimators for $\beta_{a}$ and $\Lambda_{a0}\left(t\right)$, denoted ${\hat{\beta }}_{a}$ and ${\hat{\Lambda }}_{a0}\left(t\right)$, could be obtained using the following estimating equations:

(4) $$\sum_{i=1}^{n}\int_{0}^{\infty }{\mathrm{dM}}_{i}^{\left(a,\nu \right)}\left(t\right)\theta_{D}\left(\nu \right){\bar{\theta }}_{R}\left(t\right)\mathrm{d\nu}=0,\;t\geq 0$$

(5) $$\sum_{i=1}^{n}\int_{0}^{\infty }\int_{0}^{\infty }z_{\nu }\left(t\right){\mathrm{dM}}_{i}^{\left(a,\nu \right)}\left(t\right)\theta_{D}\left(\nu \right){\bar{\theta }}_{R}\left(t\right)\mathrm{d\nu}=0,$$

where ${\mathrm{dM}}_{i}^{\left(a,\nu \right)}\left(t\right)={\mathrm{dN}}_{i}^{\left(a,\nu \right)}\left(t\right)-\exp\;\left\{\beta_{a}z_{\nu }\left(t\right)\right\}Y_{i}^{\left(a,\nu \right)}\left(t\right)d\Lambda_{a0}\left(t\right)$, and $\theta_{D}\left(l\right)$ and $\theta_{R}\left(l\right)$ are weight functions with ${\bar{\theta }}_{D}\left(t\right)=\int_{t}^{\infty }\theta_{D}\left(l\right)\mathrm{dl}$ and ${\bar{\theta }}_{R}\left(t\right)=\int_{t}^{\infty }\theta_{R}\left(l\right)\mathrm{dl}$. The estimating equations in (4) and (5) extend the equations in (10) of Yang et al.^16^ to the case where the treatment policy is a function of both treatment and discontinuation (instead of just discontinuation). If $\theta_{D}\left(\nu \right)$ is constant for all $0<\nu <T_{i}^{\left(a,\infty \right)}$, then $\hat{\beta_{a}}$ is the maximum partial likelihood estimator^16^, ^21^, ^22^ for $\beta_{a}$ and ${\hat{\Lambda }}_{a0}\left(t\right)$ is the Breslow estimator^16^, ^23^, ^24^ for $\Lambda_{a0}\left(t\right)$. Since, however, F is not observed on each individual, we approximate the solutions to (4) and (5) by solving the weighted observed‐data estimating equations

(6) $$\sum_{i=1}^{n}\omega_{\mathrm{ai}}\left(t\right)\left[{\mathrm{dN}}_{i}\left(t\right)-\lambda_{a0}\left(t\right)\exp\left\{\beta_{a}Z_{V_{i}}\left(t\right)\right\}Y_{i}\left(t\right)\mathrm{dt}\right]=0,\;t\geq 0,$$

(7) $$\sum_{i=1}^{n}\int_{0}^{\infty }\omega_{\mathrm{ai}}\left(t\right)Z_{V_{i}}\left(t\right)\left[{\mathrm{dN}}_{i}\left(t\right)-\lambda_{a0}\left(t\right)\;\exp\;\left\{\beta_{a}Z_{V_{i}}\left(t\right)\right\}Y_{i}\left(t\right)\mathrm{dt}\right]=0,$$

so that

$$d{\hat{\Lambda }}_{a0}\left(t\right)=\frac{\sum_{i=1}^{n}{\mathrm{dN}}_{i}\left(t\right)\omega_{\mathrm{ai}}\left(t\right)}{\sum_{i=1}^{n}\;\exp\;\left\{\beta_{a}Z_{V_{i}}\left(t\right)\right\}Y_{i}\left(t\right)\omega_{\mathrm{ai}}\left(t\right)},$$

and ${\hat{\beta }}_{a}$ solves

$$\sum_{i=1}^{n}\int_{0}^{\infty }\left[Z_{V_{i}}\left(t\right)-\frac{\sum_{i=1}^{n}Z_{V_{i}}\left(t\right)\exp\left\{\beta_{a}Z_{V_{i}}\left(t\right)\right\}Y_{i}\left(t\right)\omega_{\mathrm{ai}}\left(t\right)}{\sum_{i=1}^{n}\exp\left\{\beta_{a}Z_{V_{i}}\left(t\right)\right\}Y_{i}\left(t\right)\omega_{\mathrm{ai}}\left(t\right)}\right]{\mathrm{dN}}_{i}\left(t\right)\omega_{\mathrm{ai}}\left(t\right)=0,$$

where
(8)

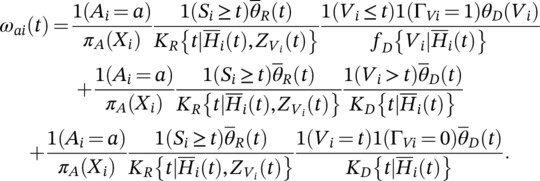

In (8), $\pi_{A}\left(X_{i}\right)=A_{i}\pi \left(X_{i}\right)+\left(1-A_{i}\right)\left\{1-\pi \left(X_{i}\right)\right\}$. Refer to the Supporting information for a proof that (6) and (7) are unbiased estimating equations for $\beta_{a}$ and $\Lambda_{a0}\left(t\right)$, respectively. We call the subject‐specific, time‐dependent weights, $\omega_{\mathrm{ai}}\left(t\right)$, inverse probability of regime compliance (IPRC) weights. Note that at any time t and for each individual i with $A_{i}=a$, only one of the three lines that make up $\omega_{\mathrm{ai}}\left(t\right)$ in (8) is nonzero. Moreover, each line in the expression for $\omega_{\mathrm{ai}}\left(t\right)$ can be broken down into three components– the fraction involving $1\left(A_{i}=a\right)$ (component 1), the fraction involving $1\left(S_{i}\geq t\right)$ (component 2), and the fraction involving $V_{i}$ (component 3). Component 1 controls for any bias that may result from not randomizing the OAC; component 2 controls for any bias resulting from artificially censoring a patient if and when he/she becomes inconsistent with the posited treatment policy; and component 3 controls for any bias resulting from the regime confounding discussed in Section 1.

The functions $\theta_{D}\left(\nu \right),{\bar{\theta }}_{R}\left(t\right),\pi \left(X_{i}\right),K_{R}\left\{t|\bar{H}\left(t\right),Z_{V_{i}}\left(t\right)\right\},f_{D}\left\{t|\bar{H}\left(t\right)\right\}$, and $K_{D}\left\{t|\bar{H}\left(t\right)\right\}$ in (8) are not known, and so they must be estimated using the observed data and plugged into (8). We write the resulting estimated TD, subject‐specific weights as ${\hat{\omega }}_{\mathrm{ai}}\left(t\right)$. Define $g\left\{\bar{H}\left(t\right)\right\}\in IR^{f}$ to be some vector‐valued function of $\bar{H}\left(t\right)$, where $f\in {\mathbb{Z}}^{+}$. Let $\alpha_{R}$ be a $\left(f+1\right)\times 1$ vector of parameters and let $\alpha_{D}$ be a f×1 vector of parameters. Suppose two separate TD Cox PH models are fit, $\lambda_{R}\left\{t|\bar{H}\left(t\right),Z_{V_{i}}\left(t\right)\right\}=\lambda_{R0}\left(t\right)\exp\left\{\alpha_{R}^{T}{\left(g{\left\{\bar{H}\left(t\right)\right\}}^{T},Z_{V_{i}}\left(t\right)\right)}^{T}\right\}$ and $\lambda_{D}\left\{t|\bar{H}\left(t\right)\right\}=\lambda_{D0}\left(t\right)\exp\left[\alpha_{D}^{T}g\left\{\bar{H}\left(t\right)\right\}\right]$, to estimate the TD hazard for regime violation and the TD hazard for treatment discontinuation, respectively. Doing so would give us estimators for the regime violation hazard and discontinuation hazard, ${\hat{\lambda }}_{R}\left\{t|\bar{H}\left(t\right),Z_{V_{i}}\left(t\right)\right\}={\hat{\lambda }}_{R0}\left(t\right)\exp\left\{{\hat{\alpha }}_{R}^{T}{\left(g{\left\{\bar{H}\left(t\right)\right\}}^{T},Z_{V_{i}}\left(t\right)\right)}^{T}\right\}$ and ${\hat{\lambda }}_{D}\left\{t|\bar{H}\left(t\right)\right\}={\hat{\lambda }}_{D0}\left(t\right)\exp\left[{\hat{\alpha }}_{D}^{T}g\left\{\bar{H}\left(t\right)\right\}\right]$, so that estimators for $K_{R}\left\{t|\bar{H}\left(t\right),Z_{V_{i}}\left(t\right)\right\}$ and $K_{D}\left\{t|\bar{H}\left(t\right)\right\}$ can be obtained by setting ${\hat{K}}_{R}\left\{t|\bar{H}\left(t\right),Z_{V_{i}}\left(t\right)\right\}=\exp\left[-\int_{0}^{t}{\hat{\lambda }}_{R}\left\{l|\bar{H}\left(l\right),Z_{V_{i}}\left(l\right)\right\}\mathrm{dl}\right]$ and ${\hat{K}}_{D}\left\{t|\bar{H}\left(t\right)\right\}=\exp\left[-\int_{0}^{t}{\hat{\lambda }}_{D}\left\{l|\bar{H}\left(l\right)\right\}\mathrm{dl}\right]$, respectively. As discussed in Yang et al.,^16^ one way to obtain a root‐*n* consistent estimator for $\theta_{D}\left(\nu \right)/f_{D}\left(\nu \right)$, using the estimators just described, would be to set $\theta_{D}\left(\nu \right)=\lambda_{D0}\left(\nu \right)\exp\left\{-\int_{0}^{\nu }\lambda_{D0}\left(l\right)\mathrm{dl}\right\}$. The estimator for $\theta_{D}\left(\nu \right)/f_{D}\left(\nu \right)$ would then be ${\hat{\theta }}_{D}\left(\nu \right)/{\hat{f}}_{D}\left(\nu \right)=\exp\left\{-\int_{0}^{\nu }{\hat{\lambda }}_{D0}\left(l\right)\mathrm{dl}\right\}/\exp\left[{\hat{\alpha }}_{D}^{T}g\left\{\bar{H}\left(t\right)\right\}\right]{\hat{K}}_{D}\left\{t|\bar{H}\left(t\right)\right\}$, which is root‐*n* consistent for $\theta_{D}\left(\nu \right)/f_{D}\left(\nu \right)$. A possible choice for the remaining weight function is to set ${\bar{\theta }}_{R}\left(t\right)=\mathrm{pr}\left(R\geq t|A,X\right)$, which can also be estimated using a Cox PH model–this time without the TD covariates. The propensity score model can be fit by logistic regression.

Choosing the weight functions in the manner described above yields stabilized weights.^15^, ^16^ Stabilized weights are desirable because they can improve the efficiency of the estimation of $\beta_{a}$, compared to when other choices for the weight function are used. See Yang et al.^16^ for a nice discussion of stabilized weights in the context of treatment regime MSMs. If the propensity model, the model for the hazard of regime violation, and the model for the hazard of treatment discontinuation are correctly specified, and ${\hat{\beta }}_{a}$ is estimated using the scheme for nuisance function estimation described above, then it can be shown that ${\hat{\beta }}_{a}$ is asymptotically Normally distributed. Yang et al.^16^ recommend using the nonparametric bootstrap^25^ to estimate the variance of ${\hat{\beta }}_{a}$.

### 3.2 Simulation study

We study the performance of the proposed IPRC weights via simulations. The simulation scenario builds upon the scenario discussed in Yang et al.^14^ Specifically, we generate the treatment indicator, A, according to a Bernoulli distribution with mean equal to 0.5, and we generate G such that $G∼\exp\left(0.2\right)$. We then simulate a 1 × 3 vector from a multivariate normal distribution with mean equal to 0.2G−4 and covariance matrix equal to $0.7^{|i-j|}$ for i,j=1,2,3. The vector represents the values of a time‐dependent covariate, $Q\left(t\right)$, at times $t_{1}=0,t_{2}=5,$ and $t_{3}=10$. We assume $Q\left(t\right)$ remains constant between times $t_{1},t_{2},$ and $t_{3}$. The time‐to‐treatment‐discontinuation, D, is generated according to the proportional hazards model $\lambda_{D}\left\{t|A,\bar{Q}\left(t\right)\right\}=0.15\exp\left\{0.15A+0.15Q\left(t\right)\right\}$. The values of the time‐dependent covariate are updated to equal $Q\left(l\right)+\log\left\{l-D\right\}$ if l>D. The time‐to‐regime‐violation is generated according to the proportional hazards model $\lambda_{R}\left\{t|A,\bar{Q}\left(t\right),{\bar{Z}}_{V}\left(t\right)\right\}=0.15\exp\left[0.15A+0.15\left\{1-Z_{V}\left(t\right)\right\}\right]$. The time‐to‐event is generated according to the proportional hazards model $\lambda_{T}\left\{t|A,\bar{Q}\left(t\right),\bar{Z}\left(t\right)\right\}=0.15\exp\left[\beta^{1*}A+\beta^{2*}\left\{1-Z_{V}\left(t\right)\right\}+\beta^{3*}A\left\{1-Z_{V}\left(t\right)\right\}+0.1Q\left(0\right)\right]$. For each observation, if R<T and A=1, the value of T associated with that observation is multiplied by 5, which means that regime violation affects failure time in one of the treatment arms. The time‐to‐censoring is generated according to the proportional hazards model $\lambda_{C}\left\{t|A,\bar{Q}\left(t\right),{\bar{Z}}_{V}\left(t\right)\right\}=0.025\exp[0.15A+0.15\{1-Z_{V}\left(t\right)]$. According to this data generating scheme, we have the following MSM: $\lambda_{\mathrm{a\nu}}\left(t\right)=\lambda_{00}\left(t\right)\exp\left[\beta^{1*}a+\beta^{2*}\left\{1-z_{\nu }\left(t\right)\right\}+\beta^{3*}a\left\{1-z_{\nu }\left(t\right)\right\}\right]$, where $\beta^{2*}+\beta^{3*}a$ quantifies the relative hazard of patients who took treatment A=a and never stopped treatment compared to those who took treatment A=a and discontinued treatment. The parameter $\beta^{3*}$ quantifies the difference in the effect of discontinuation (never stop treatment versus discontinue treatment) on the log of the relative hazard of failure had the population taken a=1, versus had the population taken a=0. Importantly, under this simulation scenario there is regime confounding because $Q\left(t\right)$ predicts discontinuation, it is affected after treatment discontinuation, and it is related to the time‐to‐event.

**FIGURE 2:**
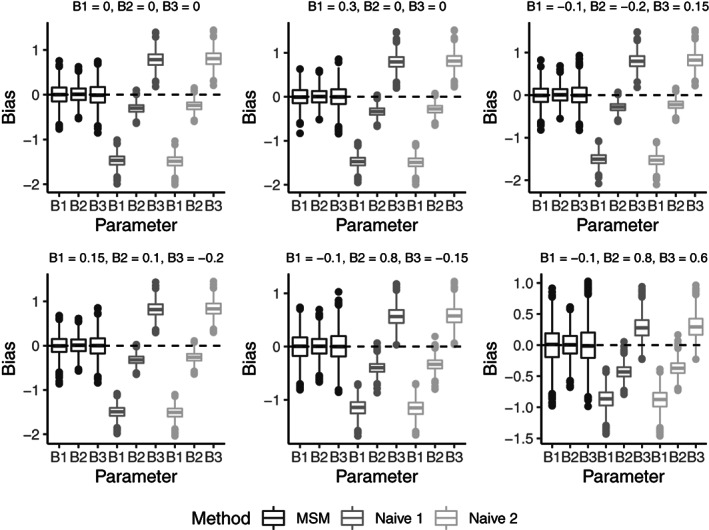
Boxplots of the parameter estimate minus the truth (i.e., $\hat{\beta_{1}^{*}}-\beta_{1}^{*}$, $\hat{\beta_{2}^{*}}-\beta_{2}^{*}$, and $\hat{\beta_{3}^{*}}-\beta_{3}^{*}$) under the three methods considered, and for each simulation scenario studied in Section 3.2

We compare three estimators for $\beta^{1*},\beta^{2*},$ and $\beta^{3*}$: (i) the estimator based on the proposed IPRC weights (treatment‐regime‐specific MSM method); (ii) the Naive 1 estimator, which is obtained by fitting a Cox PH model for failure time, adjusting for treatment, $1-Z_{V}\left(t\right)$, and the treatment × $\left\{1-Z_{V}\left(t\right)\right\}$ interaction; and iii.) the Naive 2 estimator, which is obtained by fitting a Cox PH model for failure time that adjusts for the time‐dependent covariate $Q\left(t\right)$, in addition to the covariates specified for the Naive 1 estimator. Under the 6 parameter settings considered, only our proposed treatment‐specific MSM approach consistently estimates $\beta^{1*},\beta^{2*},$ and $\beta^{3*}$ (see Table 1 and Figure 2). The naive approaches tend to underestimate $\beta^{1*}$ and $\beta^{2*}$, and they tend to overestimate $\beta^{3*}$. Moreover, the 95% Wald confidence intervals, which were generated using the robust standard error output by R software,^26^ achieve their nominal coverage under our MSM approach, but not under the naive approaches.

**TABLE 1:** Simulation results under the Naive 1 method, Naive 2 method, and our proposed treatment‐specific MSM method

Method	$\left(\beta_{1}^{*},\beta_{2}^{*},\beta_{3}^{*}\right)$	Mean Est.	SD	SE	CR
Naive 1	(0, 0, 0)	(−1.47, −0.3, 0.78)	(0.14, 0.11, 0.17)	(0.13, 0.11, 0.17)	(0, 0.2, 0.01)
	(0.3, 0, 0)	(−1.18, −0.34, 0.8)	(0.13, 0.11, 0.17)	(0.13, 0.11, 0.16)	(0, 0.12, 0)
	(−0.1, −0.2, 0.15)	(−1.61, −0.48, 0.95)	(0.14, 0.11, 0.18)	(0.13, 0.11, 0.18)	(0, 0.26, 0)
	(0.15, 0.1, −0.2)	(−1.35, −0.22, 0.62)	(0.13, 0.11, 0.17)	(0.13, 0.11, 0.17)	(0, 0.16, 0)
	(−0.1, 0.8, −0.15)	(−1.25, 0.4, 0.42)	(0.15, 0.11, 0.18)	(0.15, 0.11, 0.17)	(0, 0.06, 0.09)
	(−0.1, 0.8, 0.6)	(−0.97, 0.37, 0.88)	(0.16, 0.11, 0.18)	(0.15, 0.11, 0.18)	(0, 0.04, 0.66)
Naive 2	(0, 0, 0)	(−1.49, −0.25, 0.81)	(0.14, 0.11, 0.17)	(0.14, 0.11, 0.18)	(0, 0.42, 0)
	(0.3, 0, 0)	(−1.19, −0.28, 0.81)	(0.13, 0.11, 0.17)	(0.13, 0.11, 0.17)	(0, 0.29, 0)
	(−0.1, −0.2, 0.15)	(−1.63, −0.43, 0.98)	(0.14, 0.11, 0.18)	(0.14, 0.11, 0.18)	(0, 0.49, 0)
	(0.15, 0.1, −0.2)	(−1.36, −0.16, 0.64)	(0.13, 0.11, 0.17)	(0.13, 0.11, 0.17)	(0, 0.36, 0)
	(−0.1, 0.8, −0.15)	(−1.26, 0.46, 0.43)	(0.15, 0.12, 0.18)	(0.15, 0.12, 0.18)	(0, 0.18, 0.09)
	(−0.1, 0.8, 0.6)	(−0.98, 0.43, 0.9)	(0.16, 0.12, 0.18)	(0.16, 0.12, 0.18)	(0, 0.12, 0.64)
MSM	(0, 0, 0)	(0, 0.01, 0)	(0.22, 0.19, 0.25)	(0.22, 0.19, 0.25)	(0.95, 0.94, 0.95)
	(0.3, 0, 0)	(0.3, 0.01, 0)	(0.22, 0.19, 0.25)	(0.21, 0.19, 0.25)	(0.95, 0.94, 0.95)
	(−0.1, −0.2, 0.15)	(−0.11, −0.19, 0.15)	(0.22, 0.19, 0.26)	(0.22, 0.19, 0.26)	(0.94, 0.95, 0.95)
	(0.15, 0.1, −0.2)	(0.15, 0.11, −0.2)	(0.22, 0.19, 0.25)	(0.22, 0.19, 0.25)	(0.95, 0.95, 0.95)
	(−0.1, 0.8, −0.15)	(−0.1, 0.81, −0.15)	(0.26, 0.21, 0.28)	(0.25, 0.2, 0.27)	(0.95, 0.95, 0.95)
	(−0.1, 0.8, 0.6)	(−0.11, 0.81, 0.6)	(0.29, 0.21, 0.3)	(0.28, 0.2, 0.29)	(0.94, 0.94, 0.95)

*Note*: The mean (Mean Est.) and standard deviation (SD) of estimates of $\beta_{1}^{*}$, $\beta_{2}^{*}$, and $\beta_{3}^{*}$ are based on 2000 simulated data sets. The sample size for each simulated data set is 1000. SE is the robust standard error output from standard software. CR is the coverage rate of 95% Wald confidence intervals based on SE. Largest standard error for Mean Est., SD, and SE is 0.007, 0.005, and 0.0005 respectively.

## 4 APPLICATION TO GARFIELD‐AF STUDY

### 4.1 IPRC weight estimation

For the GARFIELD‐AF discontinuation analysis, the vector of time‐varying covariates at time l, $Q\left(l\right)$, consists of the following time‐dependent indicators that are 1 if the event is true and 0 otherwise: whether or not a patient experienced a minor bleed, major bleed, or nonmajor clinically relevant bleed since treatment initiation; whether or not a patient has had a LAAP since treatment initiation; whether or not a patient has had a nonhemorrhagicstroke/SE or since treatment initiation; and whether or not a patient has had an MI since treatment initiation. This amounts to six TD indicators–one for each event just described. When the endpoint of interest involves either nonhemorrhagic stroke/SE or MI, the corresponding TD indicators for these events are excluded from $Q\left(l\right)$. We consider 30 baseline covariates. After turning the categorical covariates into dummy variables, this amounts to 97 baseline covariates. See Table S1 for a summary of the baseline covariates.

Fitting the treatment regime MSM involves the following steps. First, we estimate $\pi \left(X_{i}\right)$ using logistic regression. We then estimate the TD hazard for regime violation described in (3) using a TD Cox PH model, and we estimate the TD hazard for discontinuation given in (2) using a TD Cox PH model with estimated TD, subject‐specific weights

(9) $${\hat{\omega }}_{\mathrm{Di}}\left(t\right)=\frac{1\left(S_{i}\geq t\right){\hat{\bar{\theta }}}_{R}\left(t\right)}{{\hat{K}}_{R}\left\{t|{\bar{H}}_{i}\left(t\right),Z_{V_{i}}\left(t\right)\right\}}=\frac{1\left(S_{i}\geq t\right){\hat{K}}_{R}\left(t|X,A\right)}{{\hat{K}}_{R}\left\{t|{\bar{H}}_{i}\left(t\right),Z_{V_{i}}\left(t\right)\right\}}.$$

In the above, and also for the computation of ${\hat{\omega }}_{\mathrm{ai}}\left(t\right)$, we choose ${\bar{\theta }}_{R}\left(t\right)=\mathrm{pr}\;\left(R\geq t|X,A\right)$. We estimate ${\bar{\theta }}_{R}\left(t\right)$ with ${\hat{K}}_{R}\left(t|X,A\right)\equiv {\hat{\bar{\theta }}}_{R}\left(t\right)$ by fitting a Cox PH model for the hazard of regime violation given just X and A. The weights in (9) serve the same purpose as piece 2 in (8). Namely, to control for any bias induced by artificially censoring patients that violated the posited treatment policy. Because the denominator in (9) is a function of the fitted TD Cox PH model for the hazard of regime violation, the TD regime‐violation model must be fit prior to fitting the TD Cox model for the discontinuation hazard. At this point, the only nuisance function that still needs to be estimated in (8) is $\theta_{D}\left(\nu \right)$. We set ${\hat{\theta }}_{D}\left(\nu \right)={\hat{\lambda }}_{D}\left(\nu |X,A\right){\hat{K}}_{D}\left(\nu |X,A\right)$, where ${\hat{\lambda }}_{D}\left(\nu |X,A\right)$ and ${\hat{K}}_{D}\left(\nu |X,A\right)$ are estimators for the discontinuation hazard and the discontinuation survival distribution given just X and A. They are also obtained by fitting a Cox PH model. Finally, the estimated TD, subject‐specific weights ${\hat{\omega }}_{\mathrm{ai}}\left(t\right)$ are computed, and $\beta_{1}^{*},$ $\beta_{2}^{*},$ and $\beta_{3}^{*}$ are estimated by fitting a TD Cox PH model with weights equal to ${\hat{\omega }}_{A_{i}}\left(t\right)=A_{i}{\hat{\omega }}_{1i}+\left(1-A_{i}\right){\hat{\omega }}_{0i}$.

All of the model fitting was done using R.^26^ For each modeling step described above, LASSO^27^ variable selection was performed to reduce the dimension of X. The Cox PH models were fit using the coxph() function in the R package survival.^28^, ^29^ Mean imputation is used to handle missingness in the continuous covariates. Inference is carried out using robust variance estimates computed by the software. Results are described in the next section.

### 4.2 Constant effect of discontinuation

**FIGURE 3:**
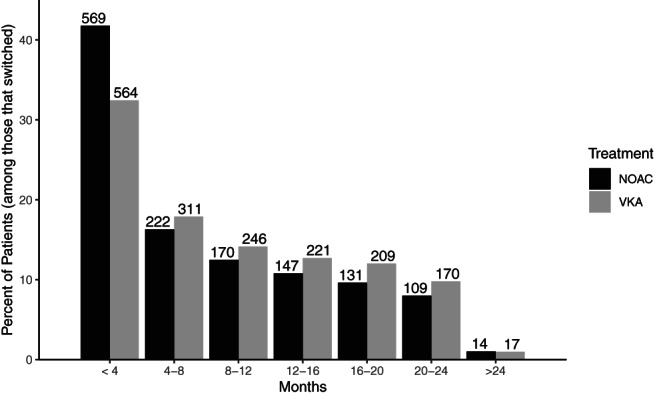
Months from start of treatment to regime violation

Of the 23,882 patients considered, 3100 patients (1738 from VKA group; 1362 from NOAC group) violated the treatment policy and were artificially censored at that time, and 365 patients (216 from VKA group; 149 from NOAC group) were censored prior to violating the treatment policy. See Figure 3 for a bar plot of when regime violation occurred for the group of patients on VKA and the group of patients on NOAC. Before discussing the results of the final MSM fit, we examine the factors associated with treatment discontinuation by looking at the results from the TD Cox PH model for the hazard of discontinuation. Variables associated with an increased hazard for discontinuation include: chronic kidney disease, history of bleeding, minor bleed during follow up, major bleed during follow up, nonmajor clinically relevant bleed during follow up, LAAP during follow up, nonhemhorrhagic stroke/SE during follow up (when not the endpoint of interest), and MI during follow up (when not the endpoint of interest). Variables associated with a decreased hazard for discontinuation include: type of atrial fibrillation, and stroke or transient ischemic attack. Refer to Table 2 for the full description of hazard ratio estimates and associated *p*‐values.

**TABLE 2:** Results from the fitted model for the hazard of treatment discontinuation

Variable	Level	HR	Lower (95%)	Upper (95%)	*P* value
Age*		0.98	0.98	0.99	<0.001
Pulse*		1.00	1.00	1.00	0.021
Type of AF (ref = new)	Paroxysmal	1.06	0.96	1.17	0.237
	Permanent	0.68	0.59	0.78	<0.001
	Persistent	0.92	0.82	1.03	0.164
SiteGR1 (ref = Asia/Europe/North America/Rest of World)	Latin America	0.64	0.49	0.83	<0.001
Country (ref = Argentina/Chile/Japan/Ukraine)	Australia	1.85	1.41	2.44	<0.001
	Austria	1.07	0.68	1.68	0.769
	Belgium	1.43	1.14	1.79	0.002
	Brazil	0.81	0.51	1.27	0.358
	Canada	0.68	0.46	1.00	0.050
	China	0.69	0.46	1.03	0.072
	Czech Republic	0.89	0.69	1.16	0.388
	Denmark	0.88	0.58	1.34	0.558
	Egypt	0.21	0.12	0.37	<0.001
	Finland	0.63	0.36	1.12	0.118
	France	0.83	0.63	1.09	0.186
	Germany	0.94	0.74	1.20	0.612
	Hungary	1.03	0.79	1.35	0.805
	India	0.17	0.08	0.34	<0.001
	Italy	1.13	0.88	1.44	0.345
	Korea	1.46	1.16	1.83	0.001
	Mexico	1.83	1.19	2.82	0.006
	Netherlands	0.56	0.40	0.79	<0.001
	Norway	0.70	0.38	1.28	0.249
	Poland	1.09	0.86	1.39	0.465
	Russia	1.34	1.06	1.70	0.016
	Singapore	1.56	0.96	2.54	0.072
	South Africa	1.88	1.42	2.48	<0.001
	Spain	1.00	0.77	1.31	0.990
	Sweden	0.70	0.51	0.97	0.031
	Switzerland	1.27	0.74	2.20	0.389
	Thailand	0.33	0.22	0.51	<0.001
	Turkey	0.76	0.55	1.06	0.107
	United Arab Emirates	0.50	0.28	0.87	0.015
	United Kingdom	1.07	0.84	1.35	0.580
	United States	1.39	1.06	1.83	0.018
Race (ref = Afro‐Caribbean/Asian (Not Chinese)/Chinese/Hispanic/Latino/Mixed/Other)	Caucasian	1.32	1.11	1.58	0.002
	Unwilling to Declare/Not Recorded	1.97	1.47	2.63	<0.001
Chronic kidney disease (ref = I/none)	II	1.17	1.04	1.31	0.009
	III	1.34	1.17	1.52	<0.001
	IV	1.57	1.13	2.20	0.008
	V	1.92	1.17	3.14	0.009
	Unknown	0.78	0.63	0.97	0.023
Care setting location (ref = anticoag clinic/thrombosis centre/hospital)	Emergency Room	1.10	0.97	1.25	0.148
	Office	0.80	0.72	0.89	<0.001
Weeks from onset to treatment*		0.96	0.93	0.99	0.005
Alcohol use (ref = abstinent)	Light	1.06	0.96	1.17	0.248
	Moderate	1.07	0.92	1.24	0.368
	Heavy	1.16	0.89	1.52	0.261
	Unknown	1.16	1.01	1.33	0.033
Coronary artery disease	Yes	1.14	1.00	1.30	0.05
Sector in which patient is treated (ref = private sector/unknown)	Public Sector	0.79	0.70	0.89	<0.001
History of bleeding	Yes	1.43	1.14	1.80	0.002
	Unknown	1.23	0.62	2.42	0.551
Stroke or TIA	Yes	0.81	0.71	0.93	0.003
Care setting specialty (ref = cardiology/geriatrics/internal medicine)	Geriatrics	0.80	0.39	1.66	0.548
	Neurology	0.70	0.48	1.02	0.066
	Primary Care/General Practice	1.19	1.05	1.36	0.006
Hypertension	Yes	0.92	0.84	1.01	0.069
	Unknown	0.72	0.36	1.44	0.351
Heart failure	Yes	0.94	0.85	1.03	0.179
Acute coronary syndrome	Yes	0.84	0.70	0.99	0.041
	Unknown	1.19	0.72	1.95	0.499
Diastolic blood pressure*		1.00	1.00	1.00	0.695
Diabetes	Yes	0.88	0.80	0.97	0.01
Dementia (ref = no/unknown)	Yes	1.17	0.82	1.66	0.401
Male	Yes	1.07	0.97	1.18	0.165
Weight (kg)*		1.00	1.00	1.00	0.698
Height (cm)*		1.00	1.00	1.01	0.204
Type of insurance (ref = combination/private (insurance))	Private (Out of Pocket)	1.29	0.96	1.72	0.093
	Public Insurance	1.06	0.93	1.20	0.386
	Unknown	0.90	0.73	1.10	0.298
Smoking status (ref = current smoker)	Ex‐Smoker	1.01	0.87	1.17	0.93
	No	1.13	0.98	1.30	0.09
	Unknown	0.94	0.76	1.14	0.52
NOAC at baseline	Yes	0.94	0.86	1.03	0.16
Systolic blood pressure*		1.00	1.00	1.00	0.83
Systemic embolism	Yes	0.79	0.47	1.32	0.37
	Unknown	1.14	0.66	1.95	0.64
Minor bleeding during F.U. period	Yes	1.90	1.61	2.23	<0.001
Major bleeding during F.U. period	Yes	10.02	7.19	13.98	<0.001
Nonmajor, clinically relevant bleeding during F.U. period		2.70	2.24	3.25	<0.001
LAAP during F.U. period	Yes	4.99	1.82	13.70	0.002
Nonhemorrhagic stroke/systemic embolism during F.U. period	Yes	4.09	2.55	6.56	<0.001
Myocardial infarction during F.U. Period	Yes	2.74	1.69	4.43	<0.001

*Note*: “HR” stands for hazard ratio. The reference group (ref) is the value “no” unless otherwise specified. ${}^{*}\mathrm{HR}$ is for a one unit increase in the variable.

**FIGURE 4:**
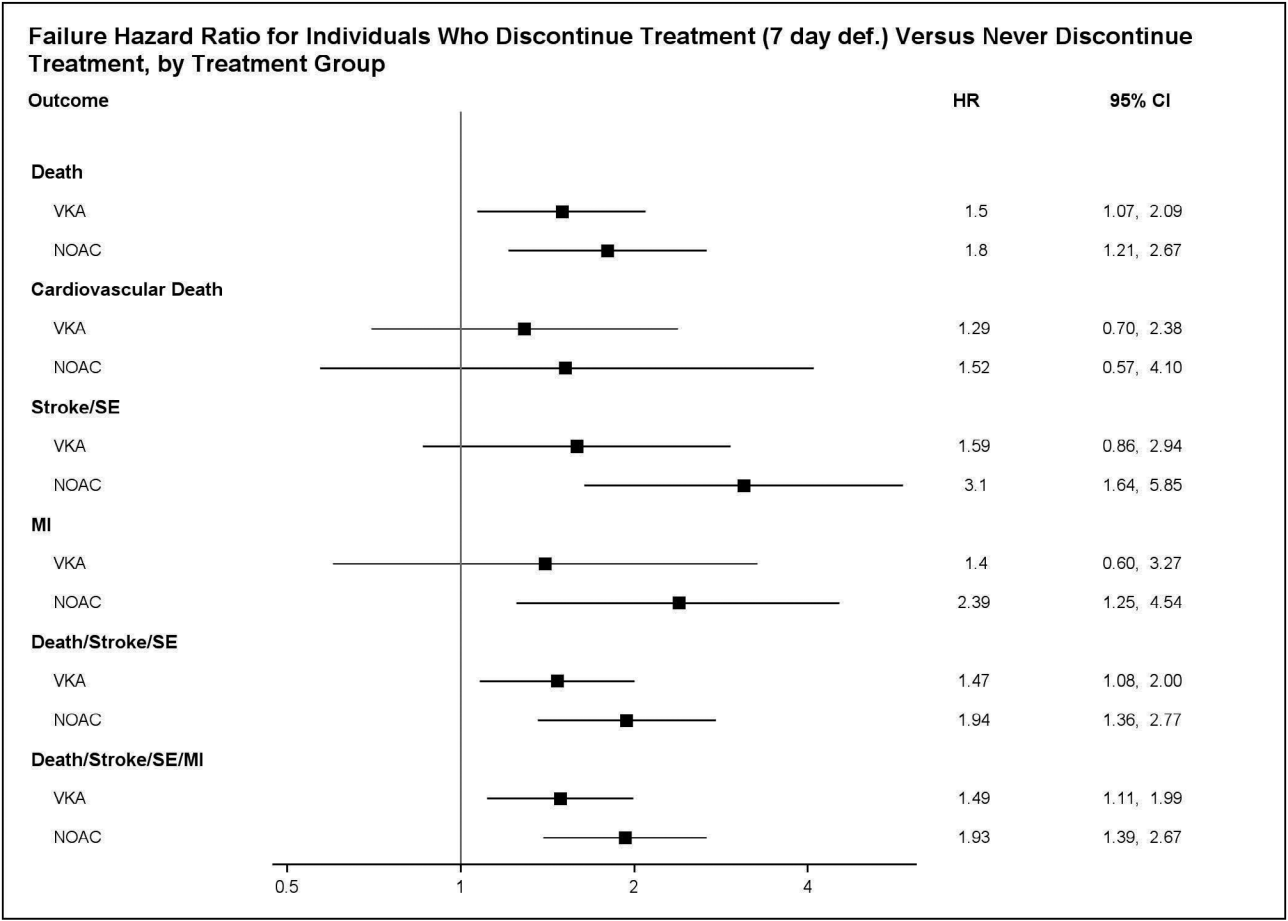
“VKA” stands for vitamin K antagonist; “NOAC” stands for non‐vitamin K oral anticoagulant; “SE” stands for systemic embolism; “MI” stands for myocardial infarction

Based on the results from fitting the final treatment regime MSM, the effect of OAC discontinuation does not significantly differ by type of OAC for the endpoints we considered (testing at α level =0.05, with smallest *p*‐value = 0.145). Refer to Table 3 for the parameter estimates and associated *p*‐values, and see Figure 4 for a forest plot of the failure hazard ratios for each endpoint had treatment been discontinued versus had treatment never been discontinued, by treatment group. Accordingly, we removed from the treatment regime MSM the interaction term for the interaction of OAC and treatment discontinuation, and we refit the model. See Table 4 for the results. We find that OAC discontinuation significantly increases the hazard for death (${\hat{\beta }}^{2*}=0.48$; *p*‐value < 0.001), stroke/SE (${\hat{\beta }}^{2*}=0.79$; *p*‐value < 0.001), MI (${\hat{\beta }}^{2*}=0.60$; *p*‐value = 0.024), death/stroke/SE (${\hat{\beta }}^{2*}=0.51$; *p*‐value < 0.001), and death/stroke/SE/MI (${\hat{\beta }}^{2*}=0.51$; *p*‐value < 0.001). After using a Bonferroni correction to adjust for multiple comparisons, there still is a significant effect of OAC discontinuation on all of the previously mentioned endpoints, except for MI.

**TABLE 3:** Results from fitting the MSM for the treatment‐specific effect of discontinuation to the GARFIELD‐AF data

Endpoint	Param.	Coef.	$\;\exp\;\left(\text{Coef}.\right)$	Robust SE	*P* value
Death	$\beta^{1*}$	−0.30	0.74	0.07	<0.001
	$\beta^{2*}$	0.40	1.50	0.17	0.018
	$\beta^{3*}$	0.18	1.20	0.27	0.496
Cardiovascular death	$\beta^{1*}$	−0.43	0.65	0.13	0.001
	$\beta^{2*}$	0.25	1.29	0.31	0.421
	$\beta^{3*}$	0.17	1.18	0.60	0.778
Stroke/SE*	$\beta^{1*}$	−0.25	0.78	0.18	0.164
	$\beta^{2*}$	0.46	1.59	0.31	0.141
	$\beta^{3*}$	0.67	1.95	0.46	0.145
MI	$\beta^{1*}$	−0.15	0.86	0.17	0.376
	$\beta^{2*}$	0.34	1.40	0.43	0.431
	$\beta^{3*}$	0.53	1.70	0.54	0.322
Death/stroke/SE*	$\beta^{1*}$	−0.29	0.75	0.07	<0.001
	$\beta^{2*}$	0.38	1.47	0.16	0.014
	$\beta^{3*}$	0.28	1.32	0.24	0.247
Death/stroke/SE*/MI	$\beta^{1*}$	−0.27	0.76	0.06	<0.001
	$\beta^{2*}$	0.40	1.49	0.15	0.007
	$\beta^{3*}$	0.26	1.29	0.22	0.248

*Note*: Results are based on the 7 day definition of discontinuation.

Abbreviations: Coef., coefficient estimate for the corresponding parameter; MI, myocardial infarction; Param., parameter; SE, standard error; SE*, systemic embolism.

**TABLE 4:** Results from fitting the MSM for the constant effect of discontinuation to the GARFIELD‐AF data

Endpoint	Param.	Coef.	$\;\exp\;\left(\text{Coef}.\right)$	Robust SE	*P* value
Death	$\beta^{1*}$	−0.28	0.76	0.07	<0.001
	$\beta^{2*}$	0.48	1.62	0.13	<0.001
Cardiovascular death	$\beta^{1*}$	−0.41	0.66	0.13	0.001
	$\beta^{2*}$	0.32	1.37	0.27	0.246
Stroke/SE*	$\beta^{1*}$	−0.14	0.87	0.16	0.392
	$\beta^{2*}$	0.79	2.21	0.23	<0.001
MI	$\beta^{1*}$	−0.08	0.92	0.16	0.600
	$\beta^{2*}$	0.60	1.83	0.27	0.024
Death/stroke/SE*	$\beta^{1*}$	−0.25	0.78	0.07	<0.001
	$\beta^{2*}$	0.51	1.66	0.12	<0.001
Death/stroke/SE*/MI	$\beta^{1*}$	−0.24	0.79	0.06	<0.001
	$\beta^{2*}$	0.51	1.66	0.11	<0.001

*Note*: Results are based on the 7 day definition of discontinuation.

Abbreviations: Coef., coefficient estimate for the corresponding parameter; MI, myocardial infarction; Param., parameter; SE, standard error; SE*, systemic embolism.

For certain endpoints (death; cardiovascular death; death/stroke/SE; death/stroke/SE/MI) there is evidence that $\beta^{1*}$ is significantly less than zero. This suggests that NOACs reduce the risk for having a clinical event, compared to VKAs, among patients who never discontinue treatment. This agrees with findings in the literature.^30^ Finally, the analyses were also run using a 30 day definition of discontinuation, in order to see how robust the results are to changes in the definition of treatment discontinuation. The results are qualitatively similar to those under the 7 day definition of treatment discontinuation, but the effect size is slightly smaller. Refer to Tables S2 and S3.

### 4.3 Time‐varying effect of discontinuation

The model we have considered thus far assumes the effect of treatment discontinuation is constant over time. To study whether there is a time‐varying effect of treatment discontinuation, we can add a term such as $\beta_{\mathrm{TD}}^{*}\left(t-\nu \right)z_{\nu }\left(t\right)$ to the current model, so that the treatment‐regime‐specific MSM becomes:

(10) $$\lambda_{\mathrm{a\nu}}\left(t\right)=\lambda_{00}\left(t\right)\exp\left\{\beta^{1*}a+\beta^{2*}z_{\nu }\left(t\right)+\beta^{3*}{\mathrm{az}}_{\nu }\left(t\right)+\beta_{\mathrm{TD}}^{*}\left(t-\nu \right)z_{\nu }\left(t\right)\right\}.$$

In this way, the effect of treatment discontinuation is now a function of the duration of treatment discontinuation. We fit the model given in (10) to the GARFIELD‐AF data, excluding the term $\beta^{3*}{\mathrm{az}}_{\nu }\left(t\right)$, as we already found that the effect of discontinuation does not significantly differ by type of OAC (see Section 4.2 and Table 3). After fitting the model to the various endpoints of interest, we find a significant time‐varying effect of treatment discontinuation for death (${\hat{\beta }}_{\mathrm{TD}}^{*}=-0.004$; *p*‐value ≤ 0.001), cardiovascular death (${\hat{\beta }}_{\mathrm{TD}}^{*}=-0.003$; *p*‐value = 0.004), death/stroke/SE (${\hat{\beta }}_{\mathrm{TD}}^{*}=-0.004$; *p*‐value ≤ 0.001), and death/stroke/SE/MI (${\hat{\beta }}_{\mathrm{TD}}^{*}=-0.004$; *p*‐value ≤ 0.001). Refer to Table 5. For each of those endpoints, ${\hat{\beta }}_{\mathrm{TD}}^{*}$ is less than zero, indicating that the effect of discontinuation on those endpoints dampens over time.

**TABLE 5:** Results from fitting the MSM for the time‐varying effect of discontinuation to the GARFIELD‐AF data

Endpoint	Param.	Coef.	$\exp\left(\text{Coef}.\right)$	Robust SE	*P* value
Death	$\beta^{1*}$	−0.29	0.75	0.07	<0.001
	$\beta^{2*}$	1.35	3.87	0.18	<0.001
	$\beta_{\mathrm{TD}}^{*}$	−0.004	1.0	0.001	<0.001
Cardiovascular death	$\beta^{1*}$	−0.42	0.66	0.13	0.001
	$\beta^{2*}$	1.11	3.03	0.33	<0.001
	$\beta_{\mathrm{TD}}^{*}$	−0.003	1.0	0.001	0.004
Stroke/SE*	$\beta^{1*}$	−0.15	0.86	0.16	0.367
	$\beta^{2*}$	1.26	3.54	0.35	<0.001
	$\beta_{\mathrm{TD}}^{*}$	−0.002	1.0	0.002	0.262
MI	$\beta^{1*}$	−0.09	0.92	0.16	0.582
	$\beta^{2*}$	0.97	2.64	0.37	0.008
	$\beta_{\mathrm{TD}}^{*}$	−0.002	1.0	0.001	0.237
Death/stroke/SE*	$\beta^{1*}$	−0.26	0.77	0.07	<0.001
	$\beta^{2*}$	1.34	3.81	0.17	<0.001
	$\beta_{\mathrm{TD}}^{*}$	−0.004	1.0	0.001	<0.001
Death/stroke/SE*/MI	$\beta^{1*}$	−0.25	0.78	0.06	<0.001
	$\beta^{2*}$	1.37	3.92	0.15	<0.001
	$\beta_{\mathrm{TD}}^{*}$	−0.004	1.0	0.001	<0.001

*Note*: Results are based on the 7 day definition of discontinuation.

Abbreviations: Coef., coefficient estimate for the corresponding parameter; MI, myocardial infarction; Param., parameter; SE, standard error; SE*, systemic embolism.

## 5 CONCLUSION

We consider a treatment‐specific marginal structural Cox model for the effect of treatment discontinuation on a survival endpoint, and we propose IPRC weights for estimating the parameters of the MSM. The IPRC weights control for three potential sources of bias–bias due to non‐randomized treatment, bias due to regime confounding, and bias caused by artificially censoring patients. The adjustment for this last source of bias, within the IPRC weights, is a key contribution of the proposed framework. Using this framework, we estimate the causal effect of OAC discontinuation in a population of patients with Atrial Fibrillation. We do not find evidence that the effect of OAC discontinuation differs by treatment (VKA/NOAC), but we do find evidence that treatment discontinuation increases the hazard for certain clinical events (death; stroke/SE; death/stroke/SE; death/stroke/SE/MI). Combining these findings with the insight that most patients that discontinue treatment do so relatively early after treatment initiation, it may be worthwhile for clinicians to emphasize the importance of remaining on OACs, especially early into treatment initiation. The proposed framework is widely applicable to other disease settings with treatment discontinuation and/or regime violation.

In the Application to GARFIELD‐AF Study, we illustrate the application of the proposed method to multiple outcomes (death; stroke/SE; death/stroke/SE; death/stroke/SE/MI). When analyzing one outcome, for example, death, we treat other variables: stroke, SE, and MI as time‐varying confounders. The strategy is useful to correct for confounding biases; however, one cannot study the effect of discontinuation on multiple outcomes simultaneously. One potential solution is to recast the problem in a competing risks setting, where the failure event is classified into one of several mutually exclusive types, and occurrence of one type of event precludes the occurrence of an event of another type. Extension of the proposed framework to competing risks is possible by changing the MSMs to event‐specific models. This will be an interesting topic for research in the future.

A limitation of this work is that it relies on a three‐part assumption of no unmeasured confounders, which is a strong yet unverifiable assumption. Additionally, we have assumed that censoring is non‐informative. If the censoring assumption is relaxed so that censoring can depend on the observed data, R can be reset to denote the time to censoring or regime violation, whichever comes first, and $\Gamma_{\mathrm{Si}}$ can be reset as the indicator that either censoring or regime violation occurred during follow up. This scenario may require a more complicated model for R than the model described in this paper. Finally, the IPRC weights that we consider are the product of three inverse probability weights–which may be unstable if the denominator(s) of those weights is(are) close to zero. In such cases, the weights may need to be truncated in order to fit the model, and sensitivity analyses should be conducted in order to examine how sensitive the results are to different levels of truncation (e.g. compare results after truncating weights greater than: 50, 100, 150). Augmenting the IPRC weighting approach using outcome regression may help, but will be a future topic for research.

## CONFLICT OF INTEREST

The authors declare no potential conflict of interest.

## Supporting information


**Table S1.** Baseline summary table for covariates considered in the GARFIELD‐AF data application.
**Table S2.** Results from fitting the MSM with $\beta^{3*}$ included in the model, under the 30 day definition of discontinuation.
**Table S3.** Results from fitting the MSM after removing $\beta^{3*}$ from the model, under the 30 day definition of discontinuation.

## Data Availability

Data are available by request due to privacy and ethical issues.

## References

[1] Wolf PA , Abbott RD , Kannel WB . Atrial fibrillation as an independent risk factor for stroke: the Framingham study. Stroke. 1991;22(8):983‐988. 1866765 10.1161/01.str.22.8.983

[2] Amin A , Houmsse A , Ishola A , Tyler J , Houmsse M . The current approach of atrial fibrillation management. Avicenna J Med. 2016;6:8. doi:10.4103/2231-0770.173580 26955600 PMC4759971

[3] Harel Z , Sholzberg M , Shah P , et al. Comparisons between novel Oral anticoagulants and vitamin K antagonists in patients with CKD. J Am Soc Nephrol. 2014;25:431‐442. doi:10.1681/ASN.2013040361 24385595 PMC3935586

[4] Chang TY , Liao JN , Chao TF , et al. Oral anticoagulant use for stroke prevention in atrial fibrillation patients with difficult scenarios. IJC Heart Vasc. 2018;20:56‐62. doi:10.1016/j.ijcha.2018.08.003 PMC6122229 30186937

[5] Hylek E , Evans‐Molina C , Shea C , Henault L , Regan S . Major hemorrhage and tolerability ofWarfarin in the first year of therapy among elderly patients with atrial fibrillation. Circulation. 2007;115:2689‐2696. doi:10.1161/CIRCULATIONAHA.106.653048 17515465

[6] Gallagher A , Rietbrock S , Plumb J , Staa T . Initiation and persistence of warfarin or aspirin in patients with chronic atrial fibrillation in general practice: do the appropriate patients receive stroke prophylaxis? J Thromb Haemostasis. 2008;6:1500‐1506. doi:10.1111/j.1538-7836.2008.03059.x 18573187

[7] Beyer‐westendorf J , Ehlken B , Evers T . Real‐world persistence and adherence to oral anticoagulation for stroke risk reduction in patients with atrial fibrillation. Europace. 2016;18:1150‐1157. doi:10.1093/europace/euv421 26830891

[8] Kakkar AK , Mueller I , Bassand JP , et al. International longitudinal registry of patients with atrial fibrillation at risk of stroke: global anticoagulant registry in the FIELD (GARFIELD). Am Heart J. 2012;163:13‐19. doi:10.1016/j.ahj.2011.09.011 22172431

[9] Thrombosis Research Institute . About GARFIELD‐AF; 2020. https://af.garfieldregistry.org/about/garfield-af

[10] Reddy VY , Holmes D , Doshi SK , Neuzil P , Kar S . Safety of percutaneous left atrial appendage closure results from the watchman left atrial appendage system for embolic protection in patients with AF (PROTECT AF) clinical trial and the continued access registry. Circulation. 2011;123(4):417‐424. 21242484 10.1161/CIRCULATIONAHA.110.976449

[11] Robins J . Marginal structural models. Proceedings of the Section on Bayesian Statistical Science, Alexandria, VA. Vol 1998. American Statistical Association; 1997:1‐10.

[12] Robins J , Hernan M , Brumback B . Marginal structural models and causal inference in epidemiology. Epidemiology. 2000;11:550‐560. doi:10.1097/00001648-200009000-00011 10955408

[13] Robins JM , Tsiatis AA . Correcting for non‐compliance in randomized trials using rank preserving structural failure time models. Commun Stat ‐ Theory Methods. 1991;20(8):2609‐2631. doi:10.1080/03610929108830654

[14] Yang S , Pieper K , Cools F . Semiparametric estimation of structural failure time model in continuous‐time processes. Biometrika. 2020;107(1):123‐136. 33162561 10.1093/biomet/asz057 PMC7646189

[15] Hernán M , Brumback B , Robins J . Marginal structural models to estimate the causal effect of Zidovudine on the survival of HIV‐positive men. Epidemiology. 2000;11:561‐570. doi:10.1097/00001648-200009000-00012 10955409

[16] Yang S , Tsiatis A , Blazing M . Modeling survival distribution as a function of time to treatment discontinuation: a dynamic treatment regime approach. Biometrics. 2018;74:900‐909. doi:10.1111/biom.12845 29359317 PMC6054909

[17] Neyman J . On the application of probability theory to agricultural experiments. Essay on principles. Section 9. Stat Sci. 1923;5:465‐472.

[18] Rubin D . Estimating causal effects of treatments in randomized and nonrandomized studies. J Educ Psychol. 1974;66(5):688‐701.

[19] Cole SR , Hernán MA . Constructing inverse probability weights for marginal structural models. Am J Epidemiol. 2008;168(6):656‐664. doi:10.1093/aje/kwn164 18682488 PMC2732954

[20] Hernán MA , Robins JM . Estimating causal effects from epidemiological data. J Epidemiol Community Health. 2006;60(7):578‐586. doi:10.1136/jech.2004.029496 16790829 PMC2652882

[21] Cox D . Regression models and life‐tables. J R Stat Soc B Methodol. 1972;34(2):187‐220. doi:10.1111/biom.12845

[22] Cox D . Partial likelihood. Biometrika. 1975;62:269‐276. doi:10.1093/biomet/62.2.269

[23] Breslow N . Discussion of the paper by D. R. Cox. J R Stat Soc Series B. 1972;34:216‐217. doi:10.1093/biomet/62.2.269

[24] Lin D . On the Breslow estimator. Lifetime Data Anal. 2008;13:471‐480. doi:10.1007/s10985-007-9048-y 17768681

[25] Efron B , Tibshirani R . An Introduction to the Bootstrap. CRC Press; 1994.

[26] R Core Team . R: A Language and Environment for Statistical Computing. R Foundation for Statistical Computing; 2020.

[27] Tibshirani R . Regression shrinkage and selection via the lasso. J R Stat Soc B Methodol. 1996;58(1):267‐288.

[28] Therneau TM , Grambsch PM . Modeling Survival Data: Extending the Cox Model. Springer; 2000.

[29] Therneau TM . *A Package for Survival Analysis in S*; 2015. version 2.38.

[30] Connolly SJ , Ezekowitz M , Yusuf S , et al. Dabigatran versus warfarin in patients with atrial fibrillation. N Engl J Med. 2009;361:1139‐1151. 19717844 10.1056/NEJMoa0905561

