# Supplementary material for: A treatment‐specific marginal structural Cox model for the effect of treatment discontinuation
Source: Pharm Stat. 2022 Mar 31;21(5):988–1004. doi: 10.1002/pst.2211 (PMC9481666; doi:10.1002/pst.2211)
Supplement: Supplementary file 1 — Table S1. Baseline summary table for covariates considered in the GARFIELD‐AF data application. Table S2. Results from fitting the MSM with β3* included in the model, under the 30 day definition of discontinuation. Table S3. Results from fitting the MSM after removing β3* from the model, under the 30 day definition of discontinuation. [file PST-21-988-s001.pdf]

Supporting Information for Treatment-Specific Marginal Structural Cox Model for the Effect of Treatment Discontinuation

The supporting information includes the proof that (6) and (7) are unbiased estimating equations and tables for the application.

1 Proof that (6) and (7) are Unbiased Estimating Equations

In order to show that (6) and (7) are unbiased estimating equations, we need to show that

$$E\left\{\begin{pmatrix} \omega_{ai}(t)[dN_i(t)-\lambda_{a0}(t)\exp\{\beta_a Z_{V_i}(t)\}Y_i(t)dt] \\ \omega_{ai}(t)Z_{V_i}(t))[dN_i(t)-\lambda_{a0}(t)\exp\{\beta_a Z_{V_i}(t)\}Y_i(t)dt] \end{pmatrix}\right\} = \begin{pmatrix} 0 \\ 0 \end{pmatrix}. \tag{1}$$

First we show that

$$\begin{aligned}
&\frac{1(V_i \le t, \Gamma_{Vi}=0)1(A_i=a)1(S_i \ge t)\overline{\theta}_R(t)}{K_D\{V_i \mid \overline{H}(t)\}K_R\{t \mid \overline{H}(t), Z_{V_i}(t)\}\pi_A(X_i)}\int_{V_i}^{t}[dN_i(t)-\lambda_{a0}(t)\exp\{\beta_a^T Z_{V_i}(t)\}Y_i(t)dt]\theta_D(v)dv \\
&+\frac{1(V_i \le t, \Gamma_{Vi}=0)1(A_i=a)1(S_i \ge t)\overline{\theta}_R(t)}{K_D\{V_i \mid \overline{H}(t)\}K_R\{t \mid \overline{H}(t), Z_{V_i}(t)\}\pi_A(X_i)}[dN_i(t)-\lambda_{a0}(t)Y_i(t)dt]\overline{\theta}_D(t) \\
&=\frac{1(V_i = t)1(\Gamma_{Vi}=0)1(A_i=a)1(S_i \ge t)\overline{\theta}_R(t)}{K_D\{V_i \mid \overline{H}(t)\}K_R\{t \mid \overline{H}(t), Z_{V_i}(t)\}\pi_A(X_i)}[dN_i(t)-\lambda_{a0}(t)\exp\{\beta_a^T Z_{V_i}(t)\}Y_i(t)dt]\overline{\theta}_D(t).
\end{aligned}$$

Proof:

$$\begin{aligned}
&\frac{1(V_i \le t, \Gamma_{Vi}=0)1(A_i=a)1(S_i \ge t)\overline{\theta}_R(t)}{K_D\{V_i \mid \overline{H}(t)\}K_R\{t \mid \overline{H}(t), Z_{V_i}(t)\}\pi_A(X_i)}\int_{V_i}^{t}[dN_i(t)-\lambda_{a0}(t)\exp\{\beta_a^T Z_{V_i}(t)\}Y_i(t)dt]\theta_D(v)dv \\
&+\frac{1(V_i \le t, \Gamma_{Vi}=0)1(A_i=a)(1(S_i \ge t)\overline{\theta}_R(t)}{K_D\{V_i \mid \overline{H}(t)\}K_R\{t \mid \overline{H}(t), Z_{V_i}(t)\}\pi_A(X_i)}[dN_i(t)-\lambda_{a0}(t)Y_i(t)dt]\overline{\theta}_D(t) \\
&=\frac{1(V_i \le t, \Gamma_{Vi}=0)1(A_i=a)1(S_i \ge t)\overline{\theta}_R(t)}{K_D\{V_i \mid \overline{H}(t)\}K_R\{t \mid \overline{H}(t), Z_{V_i}(t)\}\pi_A(X_i)}\int_{V_i}^{t}[dN_i(t)-\lambda_{a0}(t)\exp\{\beta_a^T Z_{\nu}(t)\}Y_i(t)dt]\theta_D(v)dv \\
&+\frac{1(V_i \le t, \Gamma_{Vi}=0)1(A_i=a)1(S_i \ge t)\overline{\theta}_R(t)}{K_D\{V_i \mid \overline{H}(t)\}K_R\{t \mid \overline{H}(t), Z_{V_i}(t)\}\pi_A(X_i)}[dN_i(t)-\lambda_{a0}(t)\exp\{\beta_a^T Z_{V_i}(t)\}Y_i(t)dt]\overline{\theta}_D(t) \\
&=\frac{1(V_i = t, \Gamma_{Vi}=0)1(A_i=a)1(S_i \ge t)\overline{\theta}_R(t)}{K_D\{V_i \mid \overline{H}(t)\}K_R\{t \mid \overline{H}(t), Z_{V_i}(t)\}\pi_A(X_i)}\int_{V_i}^{t}dM_i(t)\theta_D(v)dv \\
&+\frac{1(V_i = t, \Gamma_{Vi}=0)1(A_i=a)1(S_i \ge t)\overline{\theta}_R(t)}{K_D\{V_i \mid \overline{H}(t)\}K_R\{t \mid \overline{H}(t), Z_{V_i}(t)\}\pi_A(X_i)}dM_i(t)\overline{\theta_D}(t) \\
&=0+\frac{1(V_i = t, \Gamma_{Vi}=0)1(A_i=a)1(S_i \ge t)\overline{\theta}_R(t)}{K_D\{V_i \mid \overline{H}(t)\}K_R\{t \mid \overline{H}(t), Z_{V_i}(t)\}\pi_A(X_i)}[dN_i(t)-\lambda_{a0}(t)\exp\{\beta_a^T Z_{V_i}(t)\}Y_i(t)dt]\overline{\theta}_D(t) \\
&=\frac{1(V_i = t)1(\Gamma_{Vi}=0)1(A_i=a)1(S_i \ge t)\overline{\theta}_R(t)}{K_D\{V_i \mid \overline{H}(t)\}K_R\{t \mid \overline{H}(t), Z_{V_i}(t)\}\pi_A(X_i)}[dN_i(t)-\lambda_{a0}(t)\exp\{\beta_a^T Z_{V_i}(t)\}Y_i(t)dt]\overline{\theta}_D(t).
\end{aligned}$$

In the above, the first equality holds because $\exp\{\beta_a^T Z_{V_i}(t)\}=1$ when $V_i \le t$ and $\Gamma_{Vi}=0$. The second equality holds because the expression is equal to 0 except when $V_i = t$. This is because

$Y_i(t) = 0$ when $V_i < t$ and $\Gamma_{Vi} = 0$.
It follows, then, that the expression inside the expectation given in (1) can be written as

$$\begin{pmatrix} 1 \\ Z_{V_i}(t) \end{pmatrix} \frac{1(V_i \le t)1(\Gamma_{Vi} = 1)1(A_i = a)1(S_i \ge t)\overline{\theta}_R(t)\theta_D(V_i)}{f_D\{V_i \mid \overline{H}(t)\}K_R\{t \mid \overline{H}(t), Z_{V_i}(t)\}\pi_A(X_i)} \tag{2}$$

$$+ \begin{pmatrix} 1 \\ 0 \end{pmatrix} \frac{1(V_i > t)1(A_i = a)1(S_i \ge t)\overline{\theta}_R(t)\overline{\theta}_D(t)}{K_D\{V_i \mid \overline{H}(t)\}K_R\{t \mid \overline{H}(t), Z_{V_i}(t)\}\pi_A(X_i)} \tag{3}$$

$$+ \frac{1(V_i \le t, \Gamma_{Vi} = 0)1(A_i = a)1(S_i \ge t)\overline{\theta}_R(t)}{K_D\{V_i \mid \overline{H}(t)\}K_R\{t \mid \overline{H}(t), Z_{V_i}(t)\}\pi_A(X_i)} \int_{V_i}^{t} \begin{pmatrix} 1 \\ z_\nu(t) \end{pmatrix} [dN_i(t) - \lambda_{a0}(t)\exp\{\beta_a^T Z_\nu(t)\} \tag{4}$$

$$\times Y_i(t)dt]\theta_D(v)dv \tag{5}$$

$$+ \begin{pmatrix} 1 \\ 0 \end{pmatrix} \frac{1(V_i \le t, \Gamma_{Vi} = 0)1(A_i = a)1(S_i \ge t)\overline{\theta}_R(t)\overline{\theta_D}(t)}{K_D\{V_i \mid \overline{H}(t)\}K_R\{t \mid \overline{H}(t), Z_{V_i}(t)\}\pi_A(X_i)} [dN_i(t) - \lambda_{a0}(t)Y_i(t)dt]. \tag{6}$$

Refer to (2) as "component 1"; refer to (3) as "component 2"; refer to (4) and (5) as "component 3"; and refer to (6) as "component 4". We show that (1) holds by showing:

(i) The expectation of component 1 is equal to $E\left[\int_0^t \begin{pmatrix} 1 \\ z_\nu(t) \end{pmatrix} 1(T_i > v)dM_i^{(a,v)}(t)\theta_D(v)\overline{\theta}_R(t)dv\right]$.

(ii) The expectation of component 2 is equal to $E\left\{1(T_i > t)\begin{pmatrix} 1 \\ 0 \end{pmatrix} dM_i^{(a,t)}(t)\overline{\theta}_D(t)\overline{\theta}_R(t)\right\}$.

(iii) The expectation of component 3 is equal to

$$E\left\{\int_0^t 1(T_i \le \nu)\begin{pmatrix} 1 \\ z_\nu(t) \end{pmatrix} dM_i^{(a,\nu)}(t)\theta_D(\nu)d\nu\overline{\theta}_R(t)\right\}.$$

(iv) The expectation of component 4 is equal to $E\left[1(T_i \le t)\begin{pmatrix} 1 \\ 0 \end{pmatrix} dM_i^{(a,t)}(t)\overline{\theta}_D(t)\overline{\theta}_R(t)\right]$.

In the proofs below, we use "NUC" to refer to the assumption of no unmeasured confounders. We also use the following notation. Let

$$F_i = \left\{X_i, A_i, C_i, T_i, D_i, R_i, \overline{Q}_i(T_i), \overline{Z}_i(T_i), \left(D_i^{(a)}, T_i^{(a,\nu)}, R_i^{(a,\nu)}, \overline{Q}_i^{(a,\nu)}(T_i^{(a,\nu)}) : \nu < T_i^{(a,\infty)}, a \in \{0,1\}\right)\right\}$$

be the full set of potential variables, which contains all observed variables and potential outcomes for patient i. Also, let

$$F_{Ai} = \left\{X_i, C_i, T_i, D_i, R_i, \overline{Q}_i(T_i), \overline{Z}_i(T_i), \left(D_i^{(a)}, T_i^{(a,\nu)}, R_i^{(a,\nu)}, \overline{Q}_i^{(a,\nu)}(T_i^{(a,\nu)}) : \nu < T_i^{(a,\infty)}, a \in \{0,1\}\right)\right\},$$

$$F_{Di} = \left\{X_i, A_i, C_i, T_i, R_i, \overline{Q}_i(T_i), \overline{Z}_i(T_i), \left(T_i^{(a,\nu)}, R_i^{(a,\nu)}, \overline{Q}_i^{(a,\nu)}(T_i^{(a,\nu)}) : \nu < T_i^{(a,\infty)}, a \in \{0,1\}\right)\right\},$$

and

$$F_{Ri} = \left\{X_i, A_i, C_i, T_i, D_i, \overline{Q}_i(T_i), \overline{Z}_i(T_i), \left(D_i^{(a)}, T_i^{(a,\nu)}, \overline{Q}_i^{(a,\nu)}(T_i^{(a,\nu)}) : \nu < T_i^{(a,\infty)}, a \in \{0,1\}\right)\right\}.$$

Proof of (i)

$$E\left\{\frac{1(A_i=a)}{\pi_A(X_i)}\frac{1(V_i\le t,\Gamma_{Vi}=1)}{f_D\{V_i\mid X_i,\overline{Q}(t),A_i=a\}}\frac{1(S_i\ge t)}{K_R\{t\mid X_i,\overline{Q}_i(t),Z_{V_i}(t)=1,A_i=a\}}\begin{pmatrix}1\\ Z_{V_i}(t)\end{pmatrix}\right.$$

$$\left.\times[dN_i(t)-\lambda_{a0}(t)\exp\{\beta_a^TZ_{V_i}(t)\}Y_i(t)dt]\theta_D(V_i)\overline{\theta}_R(t)\right\}$$

$$=E\left[E\left\{\frac{1(A_i=a)}{\pi_A(X_i)}\frac{1(V_i\le t,\Gamma_{Vi}=1)}{f_D\{V_i\mid X_i,\overline{Q}(t),A_i=a\}}\frac{1(R_i^{(a,D_i^{(a)})}\ge t)}{K_R\{t\mid X_i,\overline{Q}_i(t),Z_{V_i}(t)=1,A_i=a\}}\begin{pmatrix}1\\ Z_{V_i}(t)\end{pmatrix}\right.\right.$$

$$\left.\left.\times[dN_i(t)-\lambda_{a0}(t)\exp\{\beta_a^TZ_{V_i}(t)\}Y_i(t)dt]\theta_D(V_i)\overline{\theta}_R(t)\mid F_{Ri}\right\}\right]\text{ by consistency}$$

$$=E\left[\frac{1(A_i=a)}{\pi_A(X_i)}\frac{1(V_i\le t,\Gamma_{Vi}=1)}{f_D\{V_i\mid X_i,\overline{Q}(t),A_i=a\}}\begin{pmatrix}1\\ Z_{V_i}(t)\end{pmatrix}[dN_i(t)-\lambda_{a0}(t)\exp\{\beta_a^TZ_{V_i}(t)\}Y_i(t)dt]\right.$$

$$\left.\times\theta_D(V_i)\overline{\theta}_R(t)E\left\{\frac{1(R_i^{(a,D_i^{(a)})}\ge t)}{K_R\{t\mid X_i,\overline{Q}_i(t),Z_{V_i}(t)=1,A_i=a\}}\mid F_{Ri},A_i=a\right\}\right]\text{ by NUC}$$

$$=E\left\{\frac{1(A_i=a)}{\pi_A(X_i)}\frac{1(V_i\le t,\Gamma_{Vi}=1)}{f_D\{V_i\mid X_i,\overline{Q}(t),A_i=a\}}\begin{pmatrix}1\\ Z_{V_i}(t)\end{pmatrix}[dN_i(t)-\lambda_{a0}(t)\exp\{\beta_a^TZ_{V_i}(t)\}Y_i(t)dt]\right.$$

$$\left.\times\theta_D(V_i)\overline{\theta}_R(t)\right\}\text{ by NUC and conistency}$$

$$=E\left[E\left\{\frac{1(A_i=a)}{\pi_A(X_i)}\frac{1(V_i\le t,\Gamma_{Vi}=1)}{f_D\{V_i\mid X_i,\overline{Q}(t),A_i=a\}}\begin{pmatrix}1\\ Z_{V_i}(t)\end{pmatrix}[dN_i^{(a,V_i)}(t)-\lambda_{a0}(t)\exp\{\beta_a^TZ_{V_i}(t)\}Y_i^{(a,V_i)}(t)dt]\right.\right.$$

$$\left.\left.\times\theta_D(V_i)\overline{\theta}_R(t)\mid F_{Ai}\right\}\right]\text{ by consistency}$$

$$=E\left[\frac{E\{1(A_i=a)\mid F_{Ai}\}}{\pi_A(X_i)}\frac{1(D^{(a)}\le t)}{f_D\{D_i^{(a)}\mid X_i,\overline{Q}(t),A_i=a\}}\begin{pmatrix}1\\ Z_{D_i^{(a)}}(t)\end{pmatrix}[dM_i^{(a,D_i^{(a)})}(t)\theta_D(D_i^{(a)})\overline{\theta}_R(t)\right]$$

by consistency

$$=E\left[E\left\{\frac{1(D^{(a)}\le t)}{f_D\{D_i^{(a)}\mid X_i,\overline{Q}(t),A_i=a\}}\begin{pmatrix}1\\ Z_{D_i^{(a)}}(t)\end{pmatrix}[dM_i^{(a,D_i^{(a)})}(t)\theta_D(D_i^{(a)})\overline{\theta}_R(t)\mid F_{Di}\right\}\right]\text{by NUC}$$

$$=E\left[E\left\{\begin{pmatrix}1\\ Z_{V_i}(t)\end{pmatrix}\frac{1(V_i\le t,\Gamma_{Vi}=1)dM_i^{(a,V_i)}(t)\theta_D(V_i)\overline{\theta}_R(t)}{f_D\{V_i\mid X_i,\overline{Q}_i(t),A_i=a\}}\mid F_{Di},A_i=a)\right\}\right]$$

by consistency and NUC

$$=E\left[\int_0^t\begin{pmatrix}1\\ z_\nu(t)\end{pmatrix}\frac{P\{V_i\in(\nu,\nu+d\nu),\Gamma_{Vi}=1\mid X_i,\overline{Q}(t),A_i=a\}}{f_D\{\nu\mid X_i,\overline{Q}_i(t),A_i=a\}}dM_i^{(a,\nu)}(t)\theta_D(v)\overline{\theta}_R(t)\right]\text{ by NUC}$$

$$=E\left[\int_0^t\begin{pmatrix}1\\ z_\nu(t)\end{pmatrix}1(T_i>v)\frac{f_D\{\nu\mid X_i,\overline{Q}_i(t),A_i=a\}}{f_D\{\nu\mid X_i,\overline{Q}_i(t),A_i=a\}}dM_i^{(a,v)}(t)\theta_D(v)\overline{\theta}_R(t)dv\right]$$

$$=E\left[\int_0^t\begin{pmatrix}1\\ z_\nu(t)\end{pmatrix}1(T_i>v)dM_i^{(a,v)}(t)\theta_D(v)\overline{\theta}_R(t)dv\right].$$

Proof of (ii)

$$E\Big\{1(V_i > t)1(A_i = a)1(S_i \geq t)\begin{pmatrix}1\\0\end{pmatrix}[dN_i(t) - \lambda_{a0}(t)\exp\{\beta_a^T \times 0\}Y_i(t)dt]\frac{\overline{\theta}_D(t)}{K_D\{t \mid X_i, \overline{Q}_i(t), A_i = a\}}$$
$$\times \frac{\overline{\theta}_R(t)}{K_R\{t \mid X_i, \overline{Q}_i(t), Z_{V_i}(t) = 0, A_i = a\}}\frac{1}{\pi_A(X_i)}\Big\}$$
$$= E\Bigg[E\Big\{1(V_i > t)1(A_i = a)1(R_i^{(a,D_i^{(a)})} \geq t)\begin{pmatrix}1\\0\end{pmatrix}[dN_i(t) - \lambda_{a0}(t)\exp\{\beta_a^T \times 0\}Y_i(t)dt]$$
$$\times \frac{\overline{\theta}_D(t)}{K_D\{t \mid X_i, \overline{Q}_i(t), A_i = a\}}\frac{\overline{\theta}_R(t)}{K_R\{t \mid X_i, \overline{Q}_i(t), Z_{V_i}(t) = 0, A_i = a\}}\frac{1}{\pi_A(X_i)} \mid F_{Ri}\Big\}\Bigg] \text{by consistency}$$
$$= E\Bigg[1(V_i > t)\frac{1(A_i = a)}{\pi_A(X_i)}\begin{pmatrix}1\\0\end{pmatrix}[dN_i(t) - \lambda_{a0}(t)\exp\{\beta_a^T \times 0\}Y_i(t)dt]\frac{\overline{\theta}_D(t)\overline{\theta}_R(t)}{K_D\{t \mid X_i, \overline{Q}_i(t), A_i = a\}}$$
$$\times E\Big\{\frac{1(R_i^{(a,D_i^{(a)})} \geq t)}{K_R\{t \mid X_i, \overline{Q}_i(t), Z_{V_i}(t) = 0, A_i = a\}} \mid F_{Ri}, A_i = a\Big\}\Bigg] \text{ by NUC}$$
$$= E\left\{1(V_i > t)\frac{1(A_i = a)}{\pi_A(X_i)}\begin{pmatrix}1\\0\end{pmatrix}[dN_i(t) - \lambda_{a0}(t)\exp\{\beta_a^T \times 0\}Y_i(t)dt]\frac{\overline{\theta}_D(t)\overline{\theta}_R(t)}{K_D\{t \mid X_i, \overline{Q}_i(t), A_i = a\}}\right\}$$

by NUC and consistency

$$= E\Bigg\{1(T_i > t)1(D_i^{(a)} > t)\frac{1(A_i = a)}{\pi_A(X_i)}\begin{pmatrix}1\\0\end{pmatrix}[dN_i^{(a,t)}(t) - \lambda_{a0}(t)\exp\{\beta_a^T \times 0\}Y_i^{(a,t)}(t)dt]$$
$$\times \frac{\overline{\theta}_D(t)\overline{\theta}_R(t)}{K_D\{t \mid X_i, \overline{Q}_i(t), A_i = a\}}\Bigg\}$$
$$= E\left[E\left\{1(T_i > t)1(D_i^{(a)} > t)\frac{1(A_i = a)}{\pi_A(X_i)}\begin{pmatrix}1\\0\end{pmatrix}dM_i^{(a,t)}\frac{\overline{\theta}_D(t)\overline{\theta}_R(t)}{K_D\{t \mid X_i, \overline{Q}_i(t), A_i = a\}} \mid F_{Ai}\right\}\right]$$
$$= E\left[\frac{E\{1(A_i = a) \mid F_{Ai}\}}{\pi_A(X_i)}1(T_i > t)1(D_i^{(a)} > t)\begin{pmatrix}1\\0\end{pmatrix}dM_i^{(a,t)}\frac{\overline{\theta}_D(t)\overline{\theta}_R(t)}{K_D\{t \mid X_i, \overline{Q}_i(t), A_i = a\}}\right]$$
$$= E\left[E\left\{1(T_i > t)1(D_i^{(a)} > t)\begin{pmatrix}1\\0\end{pmatrix}dM_i^{(a,t)}\frac{\overline{\theta}_D(t)\overline{\theta}_R(t)}{K_D\{t \mid X_i, \overline{Q}_i(t), A_i = a\}} \mid F_{Di}, A_i = a\right\}\right] \text{ by NUC}$$
$$= E\left[1(T_i > t)\begin{pmatrix}1\\0\end{pmatrix}dM_i^{(a,t)}\overline{\theta}_D(t)\overline{\theta}_R(t)E\left\{\frac{1(D_i > t)}{K_D\{t \mid X_i, \overline{Q}_i(t), A_i = a\}} \mid F_{Di}, A_i = a\right\}\right] \text{ by consistency}$$
$$= E\left[1(T_i > t)\begin{pmatrix}1\\0\end{pmatrix}dM_i^{(a,t)}\overline{\theta}_D(t)\overline{\theta}_R(t)\right] \text{ by NUC.}$$

Proof of (iii)

$$E\left\{\frac{1(V_i \leq t, \Gamma_{Vi} = 0)1(A_i = a)1(S_i \geq t)}{K_D\{V_i \mid X_i, \overline{Q}_i(t), A_i = a\}}\int_{V_i}^t \begin{pmatrix}1\\z_\nu(t)\end{pmatrix}[dN_i(t) - \lambda_{a0}(t)\exp\{\beta_a^T z_\nu(t)\}Y_i(t)dt]\right.$$
$$\left.\times\, \theta_D(\nu)d\nu\frac{\overline{\theta}_R(t)}{K_R\{t \mid X_i, \overline{Q}_i(t), Z_{V_i}(t) = 0, A_i = a\}}\frac{1}{\pi_A(X_i)}\right\}$$

$$= E\Bigg[E\Bigg\{\frac{1(V_i \le t, \Gamma_{Vi}=0)1(A_i=a)1(R_i^{(a,D_i^{(a)})} \ge t)}{K_D\{V_i \mid X_i, \overline{Q}_i(t), A_i=a\}} \int_{V_i}^t \begin{pmatrix} 1 \\ z_\nu(t) \end{pmatrix} [dN_i(t) - \lambda_{a0}(t)\exp\{\beta_a^T z_\nu(t)\} Y_i(t)dt]$$

$$\times \theta_D(\nu)d\nu \frac{\overline{\theta}_R(t)}{K_R\{t \mid X_i, \overline{Q}_i(t), Z_{V_i}(t)=0, A_i=a\}} \frac{1}{\pi_A(X_i)} \mid F_{Ri}\Bigg\}\Bigg] \text{ by consistency}$$

$$= E\Bigg[\frac{1(V_i \le t, \Gamma_{Vi}=0)}{K_D\{V_i \mid X_i, \overline{Q}_i(t), A_i=a\}} \frac{\overline{\theta}_R(t)1(A_i=a)}{\pi_A(X_i)} \int_{V_i}^t \begin{pmatrix} 1 \\ z_\nu(t) \end{pmatrix} [dN_i(t) - \lambda_{a0}(t)\exp\{\beta_a^T z_\nu(t)\} Y_i(t)dt]$$

$$\times \theta_D(\nu)d\nu E\Bigg\{\frac{1(R_i^{(a,D_i^{(a)})} \ge t)}{K_R\{t \mid X_i, \overline{Q}_i(t), Z_{V_i}(t)=0, A_i=a\}} \mid F_{Ri}, A_i=a\Bigg\}\Bigg] \text{ by NUC}$$

$$= E\Bigg\{\frac{1(V_i \le t, \Gamma_{Vi}=0)}{K_D\{V_i \mid X_i, \overline{Q}_i(t), A_i=a\}} \frac{\overline{\theta}_R(t)1(A_i=a)}{\pi_A(X_i)} \int_{V_i}^t \begin{pmatrix} 1 \\ z_\nu(t) \end{pmatrix} [dN_i(t) - \lambda_{a0}(t)\exp\{\beta_a^T z_\nu(t)\} Y_i(t)dt]$$

$$\times \theta_D(\nu)d\nu\Bigg\} \text{ by NUC}$$

$$= E\Bigg\{\frac{\overline{\theta}_R(t)1(A_i=a)}{\pi_A(X_i)} \int_0^t \frac{1(V_i \le \nu, \Gamma_{Vi}=0)}{K_D\{V_i \mid X_i, \overline{Q}_i(t), A_i=a\}} \begin{pmatrix} 1 \\ z_\nu(t) \end{pmatrix} [dN_i(t) - \lambda_{a0}(t)\exp\{\beta_a^T z_\nu(t)\}$$

$$\times Y_i(t)dt]\theta_D(\nu)d\nu\Bigg\}$$

$$= E\Bigg\{\frac{\overline{\theta}_R(t)1(A_i=a)}{\pi_A(X_i)} \int_0^t \frac{1(T_i \le \nu, \Gamma_{Vi}=0)}{K_D\{T_i \mid X_i, \overline{Q}_i(t), A_i=a\}} \begin{pmatrix} 1 \\ z_\nu(t) \end{pmatrix} [dN_i^{(a,\nu)}(t) - \lambda_{a0}(t)\exp\{\beta_a^T z_\nu(t)\}$$

$$\times Y_i^{(a,\nu)}(t)dt]\theta_D(\nu)d\nu\Bigg\}$$

$$= E\Bigg\{\frac{\overline{\theta}_R(t)1(A_i=a)}{\pi_A(X_i)} \int_0^t \frac{1(T_i^{(a,\infty)} \le \nu)1(D_i^{(a)} > T_i^{(a,\infty)})}{K_D\{T_i^{(a,\infty)} \mid X_i, \overline{Q}_i(t), A_i=a\}} \begin{pmatrix} 1 \\ z_\nu(t) \end{pmatrix} [dN_i^{(a,\nu)}(t) - \lambda_{a0}(t)\exp\{\beta_a^T z_\nu(t)\}$$

$$\times Y_i^{(a,\nu)}(t)dt]\theta_D(\nu)d\nu\Bigg\} \text{ by consistency}$$

$$= E\Bigg[E\Bigg\{\frac{\overline{\theta}_R(t)1(A_i=a)}{\pi_A(X_i)} \int_0^t \frac{1(T_i^{(a,\infty)} \le \nu)1(D_i^{(a)} > T_i^{(a,\infty)})}{K_D\{T_i^{(a,\infty)} \mid X_i, \overline{Q}_i(t), A_i=a\}} \begin{pmatrix} 1 \\ z_\nu(t) \end{pmatrix} [dN_i^{(a,\nu)}(t) - \lambda_{a0}(t)$$

$$\times \exp\{\beta_a^T z_\nu(t)\} Y_i^{(a,\nu)}(t)dt]\theta_D(\nu)d\nu \mid F_{Ai}\Bigg\}\Bigg]$$

$$= E\Bigg[\frac{E\{1(A_i=a) \mid F_{Ai}\}}{\pi_A(X_i)} \overline{\theta}_R(t) \int_0^t \frac{1(T_i^{(a,\infty)} \le \nu)1(D_i^{(a)} > T_i^{(a,\infty)})}{K_D\{T_i^{(a,\infty)} \mid X_i, \overline{Q}_i(t), A_i=a\}} \begin{pmatrix} 1 \\ z_\nu(t) \end{pmatrix} [dN_i^{(a,\nu)}(t) - \lambda_{a0}(t)$$

$$\times \exp\{\beta_a^T z_\nu(t)\} Y_i^{(a,\nu)}(t)dt]\theta_D(\nu)d\nu\Bigg]$$

$$= E\Bigg[E\Bigg\{\overline{\theta}_R(t) \int_0^t \frac{1(T_i^{(a,\infty)} \le \nu)1(D_i^{(a)} > T_i^{(a,\infty)})}{K_D\{T_i^{(a,\infty)} \mid X_i, \overline{Q}_i(t), A_i=a\}} \begin{pmatrix} 1 \\ z_\nu(t) \end{pmatrix} [dN_i^{(a,\nu)}(t) - \lambda_{a0}(t)$$

$$\times \exp\{\beta_a^T z_\nu(t)\} Y_i^{(a,\nu)}(t)dt]\theta_D(\nu)d\nu \mid F_{Di}, A_i=a\Bigg\}\Bigg] \text{ by NUC}$$

$$= E\left[E\left\{\overline{\theta}_R(t)\int_0^t \frac{1(T_i \le \nu)1(D_i^{(a)} > T_i)}{K_D\{T_i \mid X_i, \overline{Q}_i(t), A_i = a\}}\begin{pmatrix}1\\ z_\nu(t)\end{pmatrix} dM_i^{(a,\nu)}\theta_D(\nu)d\nu \mid F_{Di}, A_i = a\right\}\right] \text{ by consistency}$$

$$= E\left[\overline{\theta}_R(t)\int_0^t \begin{pmatrix}1\\ z_\nu(t)\end{pmatrix} 1(T_i \le \nu)dM_i^{(a,\nu)}\theta_D(\nu)d\nu E\left\{\frac{1(D_i > T_i)}{K_D\{T_i \mid X_i, \overline{Q}_i(t), A_i = a\}} \mid F_{Di}, A_i = a\right\}\right]$$

by consistency

$$= E\left\{\int_0^t 1(T_i \le \nu)\begin{pmatrix}1\\ z_\nu(t)\end{pmatrix} dM_i^{(a,\nu)}\theta_D(\nu)d\nu\overline{\theta}_R(t)\right\}\text{by NUC.}$$

In the above, the fifth equality holds because $dN_i^{(a,\nu)}(t) = dN_i(t)$ and $Y_i^{(a,\nu)}(t) = Y_i(t)$ for $\nu \ge T_i$.

Proof of (iv)

$$E\left\{\frac{1(V_i \le t, \Gamma_{Vi} = 0)1(A_i = a)1(S_i \ge t)}{K_D\{V_i \mid X_i, \overline{Q}_i, A_i = a\}}\begin{pmatrix}1\\ 0\end{pmatrix}[dN_i(t) - \lambda_{a0}(t)\exp\{\beta_a^T \times 0\}Y_i(t)dt]\overline{\theta}_D(t)\right.$$

$$\left.\times \frac{\overline{\theta}_R(t)}{K_R\{t \mid X_i, \overline{Q}_i(t), Z_{V_i}(t) = 0, A_i = a\}}\frac{1}{\pi_A(X_i)}\right\}$$

$$= E\left[E\left\{\frac{1(V_i \le t, \Gamma_{Vi} = 0)1(A_i = a)1(R_i^{(a,D_i^{(a)})} \ge t)}{K_D\{V_i \mid X_i, \overline{Q}_i, A_i = a\}}\begin{pmatrix}1\\ 0\end{pmatrix}[dN_i(t) - \lambda_{a0}(t)\exp\{\beta_a^T \times 0\}Y_i(t)dt]\overline{\theta}_D(t)\right.\right.$$

$$\left.\times \frac{\overline{\theta}_R(t)}{K_R\{t \mid X_i, \overline{Q}_i(t), Z_{V_i}(t) = 0, A_i = a\}}\frac{1}{\pi_A(X_i)} \mid F_{Ri}\right] \text{ by consistency}$$

$$= E\left[\frac{1(V_i \le t, \Gamma_{Vi} = 0)}{K_D\{V_i \mid X_i, \overline{Q}_i, A_i = a\}}\frac{1(A_i = a)}{\pi_A(X_i)}\begin{pmatrix}1\\ 0\end{pmatrix}[dN_i(t) - \lambda_{a0}(t)\exp\{\beta_a^T \times 0\}Y_i(t)dt]\overline{\theta}_D(t)\overline{\theta}_R(t)\right.$$

$$\left.\times E\left\{\frac{1(R_i^{(a,D_i^{(a)})} \ge t)}{K_R\{t \mid X_i, \overline{Q}_i(t), Z_{V_i}(t) = 0, A_i = a\}} \mid F_{Ri}, A_i = a\right\}\right] \text{ by NUC}$$

$$= E\left[\frac{1(V_i \le t, \Gamma_{Vi} = 0)}{K_D\{V_i \mid X_i, \overline{Q}_i, A_i = a\}}\frac{1(A_i = a)}{\pi_A(X_i)}\begin{pmatrix}1\\ 0\end{pmatrix}[dN_i(t) - \lambda_{a0}(t)\exp\{\beta_a^T \times 0\}Y_i(t)dt]\overline{\theta}_D(t)\overline{\theta}_R(t)\right]$$

by NUC and consistency

$$= E\left[\frac{1(T_i^{(a,\infty)} \le t)1(D_i^{(a)} > T_i^{(a,\infty)})}{K_D\{T_i^{(a,\infty)} \mid X_i, \overline{Q}_i, A_i = a\}}\frac{1(A_i = a)}{\pi_A(X_i)}\begin{pmatrix}1\\ 0\end{pmatrix}[dN_i^{(a,t)}(t) - \lambda_{a0}(t)\exp\{\beta_a^T \times 0\}Y_i^{(a,t)}(t)dt]\right.$$

$$\left.\times \overline{\theta}_D(t)\overline{\theta}_R(t)\right] \text{ by consistency}$$

$$= E\left[E\left\{\frac{1(T_i^{(a,\infty)} \le t)1(D_i^{(a)} > T_i^{(a,\infty)})}{K_D\{T_i^{(a,\infty)} \mid X_i, \overline{Q}_i, A_i = a\}}\frac{1(A_i = a)}{\pi_A(X_i)}\begin{pmatrix}1\\ 0\end{pmatrix} dM_i^{(a,t)}\overline{\theta}_D(t)\overline{\theta}_R(t) \mid F_{Ai}\right\}\right]$$

$$= E\left[\frac{E\{1(A_i = a) \mid F_{Ai}\}}{\pi_A(X_i)}\frac{1(T_i^{(a,\infty)} \le t)1(D_i^{(a)} > T_i^{(a,\infty)})}{K_D\{T_i^{(a,\infty)} \mid X_i, \overline{Q}_i, A_i = a\}}\begin{pmatrix}1\\ 0\end{pmatrix} dM_i^{(a,t)}\overline{\theta}_D(t)\overline{\theta}_R(t)\right]$$

$$= E\left[E\left\{\frac{1(T_i^{(a,\infty)} \le t)1(D_i^{(a)} > T_i^{(a,\infty)})}{K_D\{T_i^{(a,\infty)} \mid X_i, \overline{Q}_i, A_i = a\}}\begin{pmatrix}1\\ 0\end{pmatrix} dM_i^{(a,t)}\overline{\theta}_D(t)\overline{\theta}_R(t) \mid F_{Di}, A_i = a\right\}\right] \text{ by NUC}$$

$$= E\left[E\left\{\frac{1(T_i \le t)1(D_i > T_i)}{K_D\{T_i \mid X_i, \overline{Q}_i, A_i = a\}}\begin{pmatrix}1\\ 0\end{pmatrix} dM_i^{(a,t)}\overline{\theta}_D(t)\overline{\theta}_R(t) \mid F_{Di}, A_i = a\right\}\right] \text{ by consistency}$$

$$= E\left[1(T_i \le t)\begin{pmatrix}1\\0\end{pmatrix} dM_i^{(a,t)}\overline{\theta}_D(t)\overline{\theta}_R(t)E\left\{\frac{1(D_i > T_i)}{K_D\{T_i \mid X_i, \overline{Q}_i, A_i = a\}} \mid F_{Di}, A_i = a\right\}\right]$$
$$= E\left\{1(T_i \le t)\begin{pmatrix}1\\0\end{pmatrix} dM_i^{(a,t)}\overline{\theta}_D(t)\overline{\theta}_R(t)\right\} \text{ by NUC.}$$

By (i)-(iv), it is clear that

$$E\left\{\begin{pmatrix}\omega_{ai}(t)[dN_i(t) - \lambda_{a0}(t)\exp\{\beta_a Z_{V_i}(t)\}Y_i(t)dt]\\ \omega_{ai}(t)Z_{V_i}(t))[dN_i(t) - \lambda_{a0}(t)\exp\{\beta_a Z_{V_i}(t)\}Y_i(t)dt]\end{pmatrix}\right\}$$
$$= E\left[\int_0^t \begin{pmatrix}1\\ z_\nu(t)\end{pmatrix} 1(T_i > v)dM_i^{(a,v)}(t)\theta_D(v)\overline{\theta}_R(t)dv\right] + E\left\{1(T_i > t)\begin{pmatrix}1\\0\end{pmatrix} dM_i^{(a,t)}(t)\overline{\theta}_D(t)\overline{\theta}_R(t)\right\}$$
$$+ E\left\{\int_0^t \begin{pmatrix}1\\ z_\nu(t)\end{pmatrix} 1(T_i \le \nu)dM_i^{(a,\nu)}(t)\theta_D(\nu)d\nu\overline{\theta}_R(t)\right\} + E\left[1(T_i \le t)\begin{pmatrix}1\\0\end{pmatrix} dM_i^{(a,t)}(t)\overline{\theta}_D(t)\overline{\theta}_R(t)\right]$$
$$= E\left[\int_0^t \begin{pmatrix}1\\ z_\nu(t)\end{pmatrix} dM_i^{(a,v)}(t)\theta_D(v)\overline{\theta}_R(t)dv\right] + E\left[\begin{pmatrix}1\\0\end{pmatrix} dM_i^{(a,t)}(t)\overline{\theta}_D(t)\overline{\theta}_R(t)\right]$$
$$= \begin{pmatrix}0\\0\end{pmatrix} + \begin{pmatrix}0\\0\end{pmatrix} = \begin{pmatrix}0\\0\end{pmatrix}.$$

Therefore, (6) and (7) are unbiased estimating equations for β_a and Λ_{a0}.

2 Tables

Table S1: Baseline summary table for covariates considered in the GARFIELD-AF data application. The categorical covariate "Country" is not included in the table even though it is considered in the analysis, since there are 35 levels of this variable. "IQR" stands for interquartile range; "AC clinic" stands for anticoagulation clinic; "TIA" stands for transient ischemic attack; "VKA" stands for vitamin K antagonist; "NOAC" stands for non vitamin k oral anticoagulant.

Baseline characteristics	Patients that discontinued (n = 3114)	Patients that did not discontinue (n = 20768)
Violated the regime (%)	1437 (46.15)	1663 (8.01)
Violated the regime prior to/at discontinuation (%)	212 (6.81)	1663 (8.01)
Male (%)	1827 (58.67)	11307 (54.44)
Age, median (IQR)	70 (61, 78)	72 (64, 79)
$<$ 65 years (%)	1032 (33.14)	5257 (25.31)
65-74 years (%)	984 (31.60)	7249 (34.90)
75 years (%)	1098 (35.26)	8262 (39.78)
Race		
Caucasian (%)	2235 (71.77)	13221 (63.66)
Hispanic/Latino (%)	120 (3.85)	1321 (6.36)
Afro-Caribbean (%)	10 (0.32)	131 (0.63)
Asian (not Chinese) (%)	553 (17.76)	4796 (23.09)
Chinese (%)	51 (1.64)	504 (2.43)
Mixed/Other /Unwilling to declare (%)	145 (4.66)	795 (3.83)
Body mass index, median (IQR)	27 (24, 31)	27 (24, 31)
Missing (% missing)	677 (21.74)	4599 (22.14)
Hypertension		
Yes (%)	2377 (76.33)	16159 (77.81)
No (%)	728 (23.38)	4540 (21.86)

Continued on next page

Table S1 – *Continued from previous page*

Baseline characteristics	Patients that discontinued (n = 3114)	Patients that did not discontinue (n = 20768)
Unknown(%)	9 (0.29)	69 (0.33)
Diabetes (%)	649 (20.84)	4901 (23.6)
Smoking		
Never smoked (%)	1820 (58.45)	12356 (59.50)
Ex-smoker (%)	749 (24.05)	4675 (22.51)
Current smoker (%)	305 (9.79)	1920 (9.24)
Unknown (%)	240 (7.71)	1817 (8.75)
Alcohol consumption		
Abstinent/light (%)	2236 (71.80)	15442 (74.35)
Moderate/heavy (%)	370 (11.88)	2024 (9.75)
Unknown (%)	508 (16.31)	3302 (15.90)
Type of atrial fibrillation		
Permanent (%)	283 (9.09)	3004 (14.46)
Persistent (%)	504 (16.18)	3507 (16.89)
Paroxysmal (%)	879 (28.23)	5565 (26.80)
New (%)	1448 (46.50)	8692 (41.85)
Care setting at diagnosis		
Hospital (%)	1719 (55.20)	10935 (52.65)
Office (%)	969 (31.12)	7582 (36.51)
AC clinic/thrombosis centre (%)	9 (0.29)	99 (0.48)
Emergency room (%)	417 (13.39)	2152 (10.36)
Heart failure (%)	684 (21.97)	4650 (22.39)
Coronary artery disease (%)	672 (21.58)	4205 (20.25)
Stroke/TIA (%)	287 (9.22)	2485 (11.97)
Systemic embolization		
Yes (%)	16 (0.51)	174 (0.84)
No (%)	3078 (98.84)	20456 (98.50)
Unknown (%)	20 (0.64)	138 (0.66)
Bleeding history		
Yes (%)	88 (2.83)	338 (1.63)
No (%)	3009 (96.63)	20341 (97.94)
Unknown (%)	17 (0.55)	89 (0.43)
Chronic kidney disease		
Stage 1-2 (%)	558 (17.92)	3445 (16.59)
Stage 3-5 (%)	416 (13.36)	2198 (10.58)
None (%)	2034 (65.32)	14159 (68.18)
Unknown (%)	106 (3.40)	966 (4.65)
Acute coronary syndrome		
Yes (%)	324 (10.40)	2152 (10.36)
No (%)	2774 (89.08)	18523 (89.19)
Unknown (%)	16 (0.51)	93 (0.45)
Region		
Asia (%)	587 (18.85)	5073 (24.43)
Europe (%)	2018 (64.80)	12142 (58.46)
Latin America (%)	149 (4.78)	1621 (7.81)
North America (%)	152 (4.88)	776 (3.74)
Rest of world (%)	208 (6.68)	1156 (5.57)
Type of insurance		
Combination (%)	340 (10.92)	1805 (8.69)
Private (insurance) (%)	196 (6.29)	1343 (6.47)
Private (out of pocket) (%)	73 (2.34)	832 (4.01)
Public insurance (%)	2538 (75.72)	15688 (75.54)
Unknown (%)	147 (4.72)	1100 (5.30)
Care setting specialty		
Cardiology (%)	2033 (65.29)	13982 (67.32)
Geriatrics (%)	7 (0.22)	67 (0.32)
Internal medicine (%)	558 (17.92)	3566 (17.17)
Neurology (%)	33 (1.06)	333 (1.60)
Primary care/general practice (%)	483 (15.51)	2820 (13.58)
Dementia		
Yes (%)	36 (1.16)	293 (1.41)
No (%)	3061 (98.30)	20351 (97.99)
Unknown (%)	17 (0.55)	124 (0.60)
Sector in which patient is treated		
Private sector (%)	842 (27.04)	6018 (28.98)

Continued on next page

Table S1 – *Continued from previous page*

Baseline characteristics	Patients that discontinued (n = 3114)	Patients that did not discontinue (n = 20768)
Public sector (%)	2246 (72.13)	14583 (70.22)
Unknown (%)	26 (0.83)	167 (0.80)
Weeks from onset to treatment, median (IQR)	1.20 (0.40, 3.00)	1.5 (0.50, 3.20)
Missing (% missing)	734 (23.57)	5821 (28.03)
Pulse (IQR)	87 (72, 110)	85 (72, 105)
Mising (% missing)	189 (6.07)	1279 (6.16)
Systolic blood pressure in mm Hg (IQR)	132 (120,144.2)	132 (120, 145)
Missing (% missing)	182 (5.84)	1204 (5.80)
Diastolic blood pressure in mm Hg (IQR)	80 (70,90)	80 (70, 89)
Missing (% missing)	182 (5.84)	1204 (5.80)
Height in cm. (IQR)	168 (161, 176)	167 (160, 174)
Missing (% missing)	677 (21.74)	4599 (22.14)
Weight in kg. (IQR)	72 (57, 83)	70 (57, 82)
Missing (% missing)	677 (21.74)	4599 (22.14)
Baseline treatment		
VKA (%)	1511 (48.52)	10397 (50.06)
NOAC(%)	1603 (51.48)	10371 (49.94)

Table S2: Results from fitting the MSM for the treatment-specific effect of discontinuation to the GARFIELD-AF data. Results are based on the 30 day definition of discontinuation. "Param." stands for parameter; "Coef." contains the coefficient estimate for the corresponding parameter; "SE" stands for standard error; "SE*" stands for systemic embolism; "MI" stands for myocardial infarction.

Endpoint	Param.	Coef.	exp(Coef.)	Robust SE	Pvalue
Death					
	β^{1*}	-0.31	0.73	0.07	< 0.001
	β^{2*}	0.27	1.31	0.18	0.137
	β^{3*}	0.09	1.10	0.30	0.756
Cardiovascular Death					
	β^{1*}	-0.46	0.63	0.12	< 0.001
	β^{2*}	0.18	1.20	0.35	0.612
	β^{3*}	0.20	1.23	0.68	0.766
Stroke/SE*					
	β^{1*}	-0.24	0.79	0.17	0.168
	β^{2*}	0.54	1.71	0.33	0.102
	β^{3*}	0.35	1.41	0.47	0.465
MI					
	β^{1*}	-0.13	0.88	0.16	0.433
	β^{2*}	0.43	1.54	0.43	0.320
	β^{3*}	0.09	1.09	0.58	0.875
Death/Stroke/SE*					
	β^{1*}	-0.30	0.74	0.07	< 0.001
	β^{2*}	0.30	1.35	0.17	0.070
	β^{3*}	0.15	1.17	0.26	0.559
Death/Stroke/SE*/MI					
	β^{1*}	-0.28	0.75	0.06	< 0.001
	β^{2*}	0.33	1.39	0.16	0.036
	β^{3*}	0.11	1.12	0.25	0.660

Table S3: Results from fitting the MSM for the constant effect of discontinuation to the GARFIELD-AF data. Results are based on the 30 day definition of discontinuation. "Param." stands for parameter; "Coef." contains the coefficient estimate for the corresponding parameter; "SE" stands for standard error; "SE*" stands for systemic embolism; "MI" stands for myocardial infarction.

Endpoint	Param.	Coef.	exp(Coef.)	Robust SE	Pvalue
Death					
	β^{1*}	-0.31	0.74	0.07	< 0.001
	β^{2*}	0.31	1.37	0.15	0.032
Cardiovascular Death					
	β^{1*}	-0.45	0.64	0.13	< 0.001
	β^{2*}	0.26	1.30	0.32	0.410
Stroke/SE*					
	β^{1*}	-0.20	0.82	0.16	0.215
	β^{2*}	0.70	2.02	0.24	0.003
MI					
	β^{1*}	-0.12	0.89	0.16	0.441
	β^{2*}	0.47	1.60	0.29	0.107
Death/Stroke/SE*					
	β^{1*}	-0.29	0.75	0.06	< 0.001
	β^{2*}	0.37	1.45	0.13	0.004
Death/Stroke/SE*/MI					
	β^{1*}	-0.28	0.76	0.06	< 0.001
	β^{2*}	0.38	1.46	0.12	0.002
